# Selective isolation of extracellular vesicles from minimally processed human plasma as a translational strategy for liquid biopsies

**DOI:** 10.1186/s40364-022-00404-1

**Published:** 2022-08-07

**Authors:** Diogo Fortunato, Stavros Giannoukakos, Ana Giménez-Capitán, Michael Hackenberg, Miguel A. Molina-Vila, Nataša Zarovni

**Affiliations:** 1 Exosomics SpA, 53100 Siena, Italy; 2 Department of Genetics, University of Granada, Granada, Spain; 3 Laboratory of Oncology, Pangaea Oncology, Barcelona, Spain

**Keywords:** Extracellular vesicle, Immunoprecipitation, Liquid biopsy, Enrichment, Platelet, Plasma, Early-stage cancer

## Abstract

**Background:**

Intercellular communication is mediated by extracellular vesicles (EVs), as they enclose selectively packaged biomolecules that can be horizontally transferred from donor to recipient cells. Because all cells constantly generate and recycle EVs, they provide accurate timed snapshots of individual pathophysiological status. Since blood plasma circulates through the whole body, it is often the biofluid of choice for biomarker detection in EVs. Blood collection is easy and minimally invasive, yet reproducible procedures to obtain pure EV samples from circulating biofluids are still lacking. Here, we addressed central aspects of EV immunoaffinity isolation from simple and complex matrices, such as plasma.

**Methods:**

Cell-generated EV spike-in models were isolated and purified by size-exclusion chromatography, stained with cellular dyes and characterized by nano flow cytometry. Fluorescently-labelled spike-in EVs emerged as reliable, high-throughput and easily measurable readouts, which were employed to optimize our EV immunoprecipitation strategy and evaluate its performance. Plasma-derived EVs were captured and detected using this straightforward protocol, sequentially combining isolation and staining of specific surface markers, such as CD9 or CD41. Multiplexed digital transcript detection data was generated using the Nanostring nCounter platform and evaluated through a dedicated bioinformatics pipeline.

**Results:**

Beads with covalently-conjugated antibodies on their surface outperformed streptavidin-conjugated beads, coated with biotinylated antibodies, in EV immunoprecipitation. Fluorescent EV spike recovery evidenced that target EV subpopulations can be efficiently retrieved from plasma, and that their enrichment is dependent not only on complex matrix composition, but also on the EV surface phenotype. Finally, mRNA profiling experiments proved that distinct EV subpopulations can be captured by directly targeting different surface markers. Furthermore, EVs isolated with anti-CD61 beads enclosed mRNA expression patterns that might be associated to early-stage lung cancer, in contrast with EVs captured through CD9, CD63 or CD81. The differential clinical value carried within each distinct EV subset highlights the advantages of selective isolation.

**Conclusions:**

This EV isolation protocol facilitated the extraction of clinically useful information from plasma. Compatible with common downstream analytics, it is a readily implementable research tool, tailored to provide a truly translational solution in routine clinical workflows, fostering the inclusion of EVs in novel liquid biopsy settings.

**Supplementary Information:**

The online version contains supplementary material available at 10.1186/s40364-022-00404-1.

## Introduction

Studies focused on EVs have flourished in the last 15 years, accompanied with a persistent concern for developing, comparing and evaluating EV isolation or purification methodologies [1–11]. Most popular approaches, such as ultracentrifugation (UC) or size-exclusion chromatography (SEC), rely on physical properties to isolate particles, depending on their density or size [5, 12, 13]. Despite the major differences between UC and SEC, when purifying low complexity samples such as serum-free cell conditioned medium (CCM), where the majority of particles are indeed EVs, both techniques recover whole EVs equally well and do not enrich for particular surface phenotypes [7, 13–15]. Thus, these methodologies are extremely useful to obtain large batches of fairly pure EV samples from cell cultures and enable unbiased characterization of EV subpopulations. However, complex biosamples, particularly plasma, are composed of highly dynamic biomolecule amounts and plenty of non-vesicular particles, which outnumber actual EVs in several orders of magnitude [5, 16, 17]. Since the features of some non-vesicular particles and EVs greatly intersect (i.e. size, density or charge), UC and SEC-isolated EVs from complex biosamples contain considerable amounts of contaminants [5, 7, 18–21]. Moreover, neither isolation approach is readily compatible with routine clinical workflows, which is still an underappreciated aspect in studies attempting to harness the potential of EVs for medical diagnostics. As research narrows down, the more EVs are recognized as essential players in a variety of key biological events, stretching beyond cell communication roles, sometimes even directly promoting disease [22–30]. In addition, EVs circulate in virtually all biofluids [22, 26, 31, 32], hence their isolation or delivery can be done with minimal invasiveness. All this body of evidence opens the door to novel, high-impact scientific and technological developments, which will foster the establishment of precision medicine and next generation disease diagnostics and monitoring, through liquid biopsies. Briefly, liquid biopsies are defined as the collection of blood, and other biofluids, for the analysis of disease-specific markers or signatures. They have been widely regarded as a game changer, particularly in oncology and cancer research, holding promise for early disease detection [33–35]. Mainstream liquid biopsy strategies rely on ultra-sensitive analytical techniques to profile circulating nucleic acids, often generating large datasets that require dedicated bioinformatics pipelines to assure high precision and reproducibility. This opens the question of whether such a strategy should be termed ‘biopsy’ at all, given that the origin of harvested material is not selected. EVs bring to the liquid biopsy field the promise of selective enrichment and traceable origin, as they carry diverse macromolecular markers distinctive of tissue, cell type or condition.

Most cancer-related EV biomarker studies conducted analytical comparisons of EVs isolated in bulk, from the blood of cancer patients and control cohorts. Research has indicated that EV heterogeneity is even more pronounced than what had been previously anticipated [14, 36–42], and consequently, the paradigm is shifting as more scientists strive now to enrich for specific subpopulations. Isolation strategies based exclusively on physical properties do not enable such enrichment, evidencing the need for methodologies able to target and retrieve distinctive phenotypical characteristics of specific EV subpopulations, typically membrane proteins or other surface moieties. As such, several affinity-based EV capture approaches have been employed, making use of solid surfaces such as chips [43–47], or beads coated with antibodies [40, 48–52], aptamers [53, 54] and even peptides [55]. Still, many reports describing affinity-based methods for EV isolation fail to comprehensively address the key factor of an enrichment strategy, which is the capacity of selecting exclusively targeted subpopulations, or simply put – specificity. Besides, research articles seldom include EV spike-in models or concerns about the impact biological matrices can have on IP performance.

In the present study, we report that nano-sized superparamagnetic beads allow for direct, specific and complete immunoprecipitation (IP) of EV subpopulations from simple or complex matrices, phosphate-buffered saline (PBS) and plasma, respectively. Because EVs are small nanoparticles, we reasoned that capture efficiency could be maximized and IP reactions better controlled with similar-sized nano beads, which may bring advantages over larger ones [56]. EV immunoaffinity isolation has been vastly performed using microbeads, typically sized > 1 μm. We selected 50 nm superparamagnetic beads that allow for minimal labelling and gentle processing of target structures, whether they are carried on cells or EVs. Initially, two different surface chemistries were evaluated for their specificity and recovery efficacy. Streptavidin-conjugated beads can be coated with any biotinylated affinity reagent, conferring high versatility to this approach, which is suitable for direct and indirect IP reactions. On the other hand, "ready-to-use" beads, coated with correctly oriented, covalently-conjugated antibodies, are known for their superior specificity [57]. Despite also allowing for correct antibody orientation, we demonstrated that streptavidin-biotin-based approaches were more susceptible to non-specific interactions, ultimately resulting in lower EV IP performance, when compared to covalently-conjugated antibody bead surfaces. Recovered EV subsets could be easily quantified with a fluorescence-based immunoassay directly on beads that did not interfere with downstream processing. Due to their complex composition, inter-donor biological variation and the lack of standardized harvesting and purification methodologies, plasma samples often introduce unknown unwanted variability in downstream analytics [7, 16, 17, 42, 58, 59], which can be a major roadblock in translational research. Therefore, we also addressed understated aspects of EV IP, namely interactions between different EV phenotypes and complex plasma matrix components, which are likely relevant in other affinity-based EV isolation contexts and can compromise both recovery and specificity. Together with gene expression profiles of distinct EV subpopulations obtained from minimally processed healthy donor plasma, we concluded that IP specificity could be sustained across plasma samples from different donors. Moreover, mRNA profiles validated that different EV subpopulations were indeed recovered according to the targeted surface marker. Finally, we applied this flow to a small clinical cohort of early-stage Non-Small Cell Lung Cancer (NSCLC) patients, providing a proof of principle that emphasizes the differential clinical value extracted from distinct EV subsets. Platelet-derived EVs were identified as potentially important biomarker repositories in early-stage cancer detection.

Our optimized procedures are simple, quick, scalable and automatable, with the endpoint goal of fitting into realistically feasible clinical workflows. Nonetheless, they can also be valuable in research and development settings, when a robust enrichment of particular EV subpopulations is required, or to improve the performance of downstream assays that could potentially benefit from this pre-analytical step.

## Materials and methods

### Biological samples and patient consent

Human plasma samples for assay optimization were obtained from BioIVT (Westbury, NY, US). Whole blood was collected in K2 EDTA tubes and within 60 min post-collection, platelet-rich plasma (PRP) was obtained by centrifugation at 500 g for 10 min. PRP was further centrifuged at 1500 g for 10 min to retrieve platelet-poor plasma (PPP). A final centrifugation at 1200 g for 20 min rendered PPP into platelet-free plasma (PFP). All plasma experiments were performed with PFP.

Patient and healthy donor samples (14 vs. 14) included in the nCounter proof of principle liquid biopsy experiments derived from a prospective single-center study conducted at the Quiron Salud hospital group (Barcelona, Spain). The study was carried out in accordance with the principles of the Declaration of Helsinki under an approved protocol of the institutional review board of Quiron Salud hospital group (internal code 2021/10-ONC-DEX act no. 03/2021). Written informed consent was obtained and documented from all patients and healthy controls; samples were de-identified for patient confidentiality. Blood samples (10 mL) were collected in Vacutainer tubes (BD, Plymouth, UK). After a first centrifugation step at 120 g for 20 min, supernatants were transferred to new tubes and immediately submitted to a second centrifugation at 120 g for 5 min. Finally, supernatants were transferred to clean tubes for a third centrifugation at 360 g for 20 min. The resulting PFP was frozen at − 80 °C and used for subsequent EV IP.

### Cell culture and EV purification from cell conditioned media (CCM)

Characterized EV spike-in models were produced as previously described [15]. Briefly, human cell lines HT29, A549, 22RV1 (ATCC) and HEK293-pRTS-CA9 (courtesy of prof. Dr. Reinhard Zeidler, Helmholtz Zentrum München, Germany) were grown in complete medium supplemented with 10% FBS (Euroclone) and 1% pen/strep (Sigma). McCoy’s 5A medium (Invitrogen) was used for HT29, DMEM (Euroclone) for HEK293 and A549 and RPMI*-*1640 (Euroclone) for 22RV1. Cells were expanded in T75 and T150 flasks, under a humid atmosphere of 5% CO2 at 37 °C. At 70% confluence, cells were washed 2 times with 1x PBS and conditioned in serum-free medium for 48-72 h. CCM was harvested and pre-cleared by differential centrifugation at 300 g for 10 minutes, 1200 g for 20 minutes, and 10000 g for 30 minutes at 4 °C. Pre-cleared CCM was stored at − 80 °C.

For EV purification, pre-cleared CCM was concentrated by ultrafiltration (Amicon® Stirred Cell, Ultracel 100 kDa Ultrafiltration Discs, Merck Millipore), from a maximum volume of 500 mL down to 10 mL. Concentrated CCM was purified by SEC (qEV10 35 nm, Izon Science), pre-equilibrated with 1 × 0.22 μm filtered PBS. Briefly, as 10 mL of concentrated CCM were loaded in column, the eluate was immediately collected in 1 mL fractions. EV-containing fractions were pooled (16 to 40, pool volume ≈24-25 mL) and concentrated down to 0,5-1 mL by 100 kDa ultrafiltration (Amicon® Ultra-15 Centrifugal Filter Unit, Merck Millipore). Purified EVs were aliquoted and stored at − 80 °C. Experiments were added to the EV-TRACK knowledgebase (EV-TRACK ID: EV220314).

### EV staining protocols

To generate traceable and easily quantifiable EV spikes for IP recovery experiments, purified EVs were stained with CellTrace™ CFSE (Thermo Fisher Scientific). EV concentration was determined by nano flow cytometry (nFCM). Each staining reaction contained 2 × 10^9^ EVs (10^8^ particles/μL) and was incubated with 10 μM CFSE for 1h 30’ at 37 °C. After pooling up to 6 staining reactions, excess CFSE was eliminated by SEC. The volume of pooled staining reactions was adjusted with filtered PBS up to 500 μL, which were loaded in SEC columns (qEVoriginal 35 nm, Izon Science) and the eluate immediately collected. The first 3 mL were discarded and the following 1,5 mL collected in a clean tube, according to the EV elution profile. Size and concentration of CFSE-labelled EVs was re-analysed by nFCM. CFSE-stained spikes were always freshly prepared and measured before each experiment. Endogenous staining of 22RV1 EVs was carried out using an amphipathic near infra-red (NIR) fluorescent probe (kindly provided by prof. Dr. Donal O’Shea, RCSI, Dublin, Ireland), which is effectively internalized by cells, spreads through the cytoplasm and becomes stably incorporated in secreted EVs [60]. At 70% of confluence, 22RV1 cells were incubated in complete medium, supplemented with NIR (5 μM) for 2 h at 37 °C, under a humid atmosphere of 5% CO2. NIR-supplemented medium was discarded and cells washed three times with PBS to eliminate all traces of unincorporated dye. Cells were conditioned in serum-free medium for 48-72 h and CCM was collected for the purification of NIR-labelled EVs, following the procedures described in the section above. EV Surface protein profiling was performed by staining with fluorescently-tagged primary antibodies. Similarly, 2 × 10^9^ EVs were incubated with antibodies for 1 h at 37 °C. Excess unbound antibodies were washed off in three rounds of buffer exchange with filtered PBS on 500 μL ultrafiltration spin columns (Nanosep® 300 kDa Centrifugal Devices, Pall Corporation).

### Nano flow cytometry: instrument setup and EV analysis

We employed a dedicated nFCM platform (Flow NanoAnalyzer, nanoFCM Inc.) that enables single particle analysis in sheathed flow, for the characterization of EVs between 40 and 200 nm. This system featured three independent single-photon counting modules, which recorded side scatter (trigger channel) and fluorescence signals for each particle that crossed the instrument’s interrogation zone and could be excited with a focused 488 nm laser beam. The instrument was aligned and calibrated at each run with size and concentration standard beads (nanoFCM Inc.). Samples and blanks (filtered PBS) were read at a constant pressure of 1 kPa for 1 min and at a maximum event rate of 12k events/min to avoid swarm effects [61, 62] in EV detection. Between samples, the instrument was cleaned with 1x cleaning solution (nanoFCM Inc.) and the capillary rinsed with ultrapure water. Fluorescence thresholds were set based on unstained EV samples and blanks. Background fluorescence stemming from the presence of unbound free dyes resulted in elevated thresholds. Such samples were either further washed or excluded from the study, to ensure accurate detection of fluorescent events. Dot plots were generated using the NF Profession 1.0 software (nanoFCM Inc.), required also to operate the system. On the Y axis, fluorescence intensity was plotted as peak area and on the X axis featured the peak height of side scatter (SSC) values.

### Bulk fluorescence measurements

For direct IP readouts, 100 μL of bead samples were loaded in black opaque 96-well plates (PerkinElmer) and read in a fluorometric plate reader (CLARIOstar Plus, BMG Labtech). Optimal gain and focal height settings were adjusted to the brightest wells. A total of 81 fluorescence measurements were acquired throughout the entire area of each well (9 × 9 data point matrix scan). The average value of all measurements was considered per well. As beads did not interfere with measurements, nor contribute to background fluorescence, raw sample fluorescent signals were normalized to filtered PBS and data is presented as signal-to-noise (S/N) or as % of input. For indirect IP readouts, 200μL of the IP flow-through were measured in parallel with input controls (IPs lacking beads). As described above, settings were adjusted to the brightest wells, which in this case were always input controls. Indirect data points were presented as percentage of recovered input, determined as:

$$\% of\ input\ (flowthrough)=1-\frac{IP\ flowthrough\ (S/N)}{input\ (S/N)}$$

### IP reactions and in-column fluorescent staining

IP reactions were conducted in 0,22 μm filtered PBS-BSA 0,1% w/v and human plasma, hereon appointed as simple and complex matrices, respectively. EV spikes contained between 5 × 10^6^ and 5 × 10^8^ particles, to which superparamagnetic antibody-conjugated MACS beads (Miltenyi Biotec) were added in excess (1 to 5 μL), since bead concentration could be accurately quantified by nFCM. As methodologies for absolute quantification of true plasma-derived EVs are still lacking, we confirmed that the number of beads applied for IPs in complex matrices allowed for maximum recovery.

Streptavidin MACS beads (Miltenyi Biotec) were coated with 2 μg of biotinylated antibody per 20 μL of beads under agitation, for 30 min at RT. To eliminate antibodies in excess, antibody-coated streptavidin beads were loaded in magnetized pre-equilibrated MACS μColumns (Miltenyi Biotec), washed 3x with 200 μL of PBS-Tween20 0,1% v/v and 2x with 400 μL of PBS. Columns were removed from the magnet, placed on clean collection tubes and beads eluted in 100 μL of PBS, with plungers. Concentration was measured by nFCM. For precise comparisons, the number of antibody-coated streptavidin MACS beads applied per IP was matched to the number of covalently-conjugated ones.

Complete IP reactions were incubated for 1 h at RT under agitation. Subsequently, IPs were loaded in magnetized pre-equilibrated MACS μColumns, washed 3x with 200 μL of PBS-Tween20 0,1% v/v and 2x with 400 μL of PBS. Then, columns were de-magnetized and the bead-EV complexes eluted in 100 μL of PBS in clean collection tubes. Additionally, bead-bound recovered EVs could also be stained with fluorescently-labelled primary antibodies, directly inside μColumns. Firstly, antibody master mixes were prepared in PBS, containing antibodies at optimized concentrations. Then, 40 μL of mix were run through magnetized columns containing washed bead samples. Additional 20 μL were added to ensure the void volume of columns was flooded in antibody staining mix. At this point, columns could be de-magnetized and incubated for 1 h at RT, protected from light. Stained bead samples were placed back on the magnet and washed 3x with 200 μL of PBS-Tween20 0,1% v/v and 2x with 400 μL of PBS. With de-magnetized columns on top of clean collection tubes, stained bead samples were eluted in 100 μL of PBS.

### RNA extraction

Bead sample volume was adjusted to 250 μL with PBS, to which 750 μL of TRIzol™ LS (Thermo Fisher Scientific) were added. Samples were vortexed for 30s and incubated for 10 min at RT. 200 μL of chloroform were added, tubes shaken for 30s and incubated for 5 min at RT. Phases were separated by centrifugation for 15 min at 12000 g and 4 °C and the aqueous phase was transferred to a clean tube,  to which 2.5 μL of RNA-grade glycogen (20 mg/mL, ThermoFisher Scientific) and 500 μL of isopropanol were added. Samples were incubated for 10 min at RT and centrifuged for 10 min at 12000 g and 4 °C. Supernatants were discarded and 1 mL of a 75% ethanol solution was added to wash RNA pellets. Tubes were vortexed briefly and centrifuged for 5 min at 7500 g and 4 °C. Supernatants were discarded and RNA pellets air-dried for 5 min. RNA pellets were resuspended in 10 μL of nuclease-free water. To 10 μL of RNA sample, 1 μL of DNA digestion buffer and 1 μL of DNase I (Zymo Research) were added. Samples were mixed, spun down and incubated for 15 min at RT. For DNase inactivation, EDTA was added at 50 mM and samples incubated for 10 min at 65 °C. RNA samples were cooled at RT and stored at − 80 °C.

### Cryogenic transmission electron microscopy (Cryo-TEM)

For cryo-sample preparation, 2.3 μL of the sample were applied to Quantifoil holey carbon grids (copper Multi A, Quantifoil Micro Tools GmbH) that were previously glow discharged. Excess fluid was blotted from the grid and plunge frozen in liquid ethane using a FEI Mark IV plunge freezer to achieve sample vitrification. Frozen samples were stored in liquid nitrogen until EM imaging in a Philips CM200FEG microscope equipped with a TVIPS TemCam-F224HD CCD camera and a Gatan 626 Cryo-Holder.

### ddPCR

One-step ddPCR reaction master mixes were prepared considering a volume of 20 μL per sample. Briefly, master mixes contained 1x Supermix, 20 U/μL of reverse transcriptase and 15 mM of DDT (One-Step RT-ddPCR Advanced Kit for Probes, BioRad). Gene expression ddPCR reactions were performed in a duplex configuration, using commercially available assays to amplify GAPDH and carbonic anhydrase 9 (CA9) (Assay IDs: dHsaCPE5031597; dHsaCPE5055974, Bio-Rad), containing HEX and FAM-conjugated reporter probes, respectively. Both assays were diluted 1:20 in the master mix. For each sample, 5 μL of RNA was thoroughly mixed with master mix and 20 μL were transferred to DG8™ Cartridges (Bio-Rad). Positive and no-template controls were included in each run. Next, 70 μL of Droplet Generation Oil (Bio-Rad) were loaded in the cartridge, which was then placed inside the QX200™ Droplet Generator (Bio-Rad). Droplets were generated and transferred to 96-well PCR plates (suppl). Plates were sealed with heat seal foil (Bio-Rad) on a PX1 PCR Plate Sealer (Bio-Rad) at 180 °C for 5 sec. Sealed 96-well plates were inserted in a T100 Thermal Cycler (Bio-Rad) and amplification conditions set as follows: Reverse transcription was performed at 42 °C for 60 min, followed by an enzyme activation step at 95 °C for 10 min and 39 cycles of denaturation at 95 °C for 30 sec and extension at 55 °C for 1 min, with a ramp rate of 3 °C/sec. A final step of 98 °C for 10 min deactivated the enzyme and amplified products were kept at 4 °C. Droplets were read in a QX200 Droplet Reader (Bio-Rad) and gene expression data analysed in QuantaSoft™ Version 1.7 (Bio-Rad).

### Magnetic beads and antibodies

Tables 1 and 2  summarize the affinity reagents used in this study:

**Table 1:** Magnetic beads (Miltenyi Biotec)

Beads	Product code	Species and Isotype
Streptavidin MicroBeads	130–048-101	N/A
Exosome Isolation Kit CD9, human	130–110-913	Mouse, IgG1
CD61 MicroBeads, human	130–051-101	Mouse, IgG1
Anti-PE MicroBeads UltraPure	130–105-639	Mouse, IgG1
Exosome Isolation Kit Pan, human	130–110-912	Mouse, IgG1

**Table 2:** Primary antibodies (Exbio Praha, a.s)

Antibody	Final concentration (μg/mL)	Product code
Anti-Human CD9 Alexa Fluor® 488	5,2	A4–208-T100
Anti-Human CD9 PE	4	1P-208-T100
Anti-Human CD41 PE	2	1P-309-T100
Mouse IgG1 Isotype Control PE	2	1P-632-C100
Anti-Human CD9 Biotin	stock at 1000	1B-208-C100
Mouse IgG1 Isotype Control Biotin	stock at 1000	1B-632-C100

### Statistical tests and specificity

Experimental datapoints were obtained in triplicate, unless stated otherwise. Mean values were plotted with standard deviation error bars throughout. Independent variables were compared and *p*-values obtained through unpaired, two-tailed Student’s t-tests, assuming equal variance. Statistical comparisons that output *p-*values ≥0.05 were deemed non-significant (ns). All the statistically significant comparisons were labelled according to *p-*values: * for *p* < 0.05; ** for *p* < 0.01; *** for *p* < 0.001 and **** for *p* < 0.0001. Prism 9.1.1 (GraphPad Software) was used for the visualization and presentation of data. Specificity was calculated as: $1-\frac{average\ S/N\ \left( negative\ control\right)}{average\ S/N\ (target).}$

### NanoString nCounter sample processing

DNAse-treated RNA samples were converted to cDNA using the nCounter® Low RNA Input Kit (NanoString Technologies), following instructions provided by the supplier. Briefly, 4 μL of RNA was used for cDNA conversion, which was followed by a pre-amplification step consisting on 14 cycles of target-specific PCR amplification using the Human Immunology V2 Primer Pool (NanoString Technologies), according to the gene expression panel analysed later on. Hybridization reactions were prepared using the Reporter CodeSet and Capture ProbeSet regents from the nCounter Human Immunology v2 Panel (NanoString Technologies) and carried out for 18 h at 65 °C. This panel includes a total of 594 genes, 579 of them involved in the immune response and 15 commonly used reference control genes. It contains also spikes of synthetic DNA targets at varying concentrations and 8 ERCC RNAs that function as internal positive and negative controls, respectively. Hybridized samples were processed in the nCounter® FLEX Analysis System (NanoString Technologies). Data was exported in RCC files for further analysis.

### Data normalisation and differential expression analysis

Raw data was exported in RCC-formatted files using the nSolver Analysis Software (version 4.0.70, NanoString Technologies). Pre-processing, normalisation, and downstream exploratory and differential expression (DE) analyses were carried out with R (version 4.0.3). Each single Nanostring run (12 samples per run) was defined as one batch. Essentially, NanoStringQCPro (version 1.22.0) package was utilised to import raw RCC files into the R environment and to perform an initial assessment of data quality and integrity. More precisely, the performance of NanoString standard Imaging, Binding Density, Positive Control Linearity, and Limit of Detection quality control metrics was examined for potential outlier samples. Next, samples were subjected to various exploratory analyses for thorough data examination. Boxplots, correlation heatmaps, principal component analysis (PCA), multidimensional scaling (MDS) and other plots were created using the ggplot2 (version 3.3.5), pheatmap (version 1.0.12), and ggpubr (version 0.4.0) packages. Moreover, the interquartile range (1.5 IQR rule) was used to detect, mark and remove potential outlier samples. Pre-processing of the data was then performed gene and sample-wise. In order to remove lowly expressed genes with excess background noise, several filtering steps were used. Firstly, the edgeR (version 3.32.1) function filterByExpr was used to remove lowly expressed genes. One additional function based on the negative control (NC) sequences was also used to filter out lowly expressed genes. Specifically, the median value of the 8 NC sequences was calculated for each sample and subtracted from the endogenous genes. After the transformation using NC sequences, genes that fell below 0 in more than 30% of the samples were removed from further analysis. Assessment of the various filtering steps was concluded again by MDS and PCA plots. Normalisation of the raw filtered data and DE analysis was attained using the DESeq2 (version 1.30.1) package with default parameters. Standard relative log expression (RLE) and PCA plots were used to evaluate the performance of the normalisation before proceeding with the differential expression analysis. Finally, fold change and adjusted *p*-values obtained from DE analyses were log2 and log10 transformed, respectively, and results visualised on volcano plots. A fold change of 2 and adjusted *p*-value of 0.05 were set as DE thresholds.

## Results

### Antibody-conjugated beads outperform antibody-coated streptavidin beads for EV IP, in simple and complex matrices

Initially, streptavidin-conjugated beads, coated with biotinylated antibodies (MACS-STV), were compared against beads directly coated with covalently-conjugated antibodies (MACS). The performance of both bead surfaces was tested in the context of EV IP by assessing the recovery of fluorescently-labelled HEK293 EVs in PBS-BSA, with anti-CD9 and negative control antibody-coated beads. Isotype antibody-coated beads served as negative control to evaluate the specificity of MACS-STV, while anti-CD61-conjugated beads (MACS-CD61) played the same role for MACS. Since CD61 represents a cluster of differentiation of the platelet lineage, and was absent from our cell lines and respective EVs, it represented an appropriate negative control for cell line-derived EV IPs. In this case, traceable EV spike-in models were generated by staining HEK293 EVs with CFSE, which resulted in 87,9% of fluorescently-labelled particles, as detected by nFCM (Sup. Fig. 1A). Bead concentration was also measured to assure that the number of beads outnumbered fluorescent EV inputs in this and in the following experiments (Sup. Table 1).

**Fig. 1:**
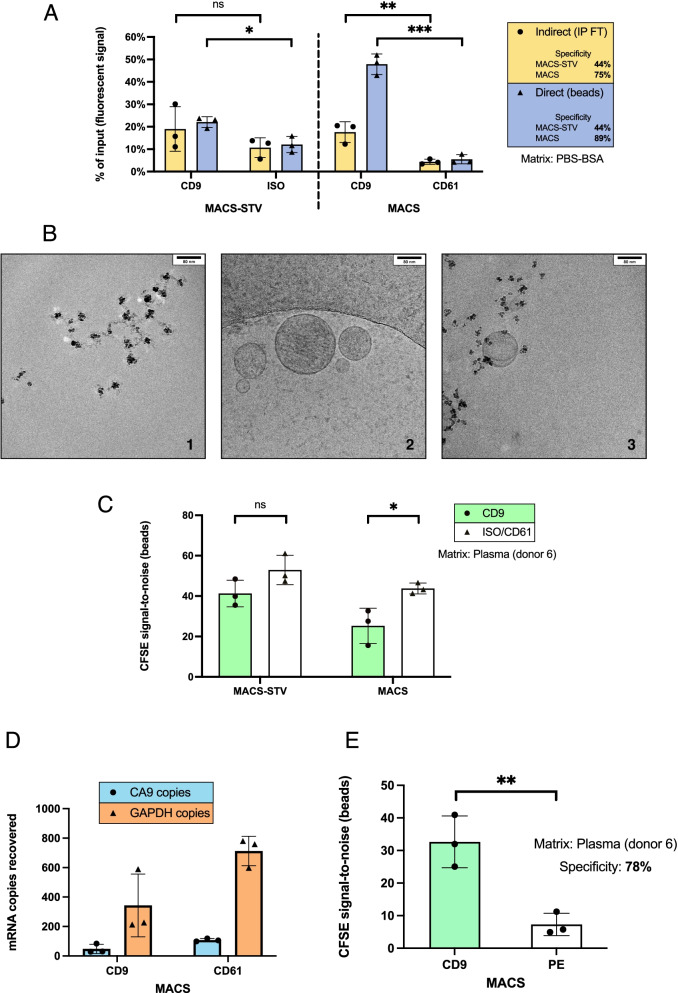
Differential recovery of EV subpopulations in simple and complex matrices, between streptavidin-coated and covalently-conjugated beads. (**A**) CFSE-stained HEK293 EVs were isolated from PBS-BSA with MACS-STV and MACS. CD9 was the IP target and ISO/CD61 were negative controls. Recovery was plotted as % of input, obtained using fluorescent signals of samples and input. Fluorescence of IP flow-throughs (FT) allowed for an indirect calculation of recovery (yellow), whereas fluorescence on beads provided a direct recovery measure (blue). Specificity represents the difference (in percentage) between target and negative control signal. (**B**) Cryo-TEM images of triple-coated MACS beads (1), HEK293 EVs (2) and the IP complex formed between both in PBS-BSA (3). Respective scale bars are shown on the top right corner of each image. (**C**) S/N ratios of CFSE-stained HEK293 EVs recovered from plasma (donor 6) on MACS-STV and MACS. The IP target was CD9 and the negative controls were ISO/CD61. (**D**) After RNA isolation from MACS CD9 and CD61 samples obtained in (C), GAPDH and CA9 mRNA copies were measured by ddPCR. (**E**) S/N ratios of CFSE-stained HEK293 EVs recovered from plasma (donor 6) on MACS beads, coated with anti-CD9 and anti-PE antibodies. IP specificity was 78%

CFSE-stained HEK293 EVs were incubated with beads and fluorescent readouts acquired for direct (beads) and indirect (IP flow-through) estimations of IP recovery. MACS-STV achieved 44% of specificity, regardless of the readout, whereas with MACS, 89% of specificity was observed by direct measurement and 75% by indirect measurement (Fig. 1A). Additionally, the fluorescent signal detected in MACS-CD9 beads evidenced a 48% recovery of CFSE-HEK293 input, which coincides with an average CD9 expression of 42% in these EVs (Sup. Fig. 1B). Considering a 5,5% of non-specific pull-down signal by MACS-CD61, nearly 100% of the CD9 subpopulation was efficiently recovered. Instead, direct measurements on MACS-STV revealed that 22,1% and 12,1% of spike was recovered with anti-CD9 and isotype control-coated beads, respectively. These results indicate that MACS but not MACS-STV, enabled IP of the entire CD9-positive subpopulation in PBS-BSA.

**Fig. 2:**
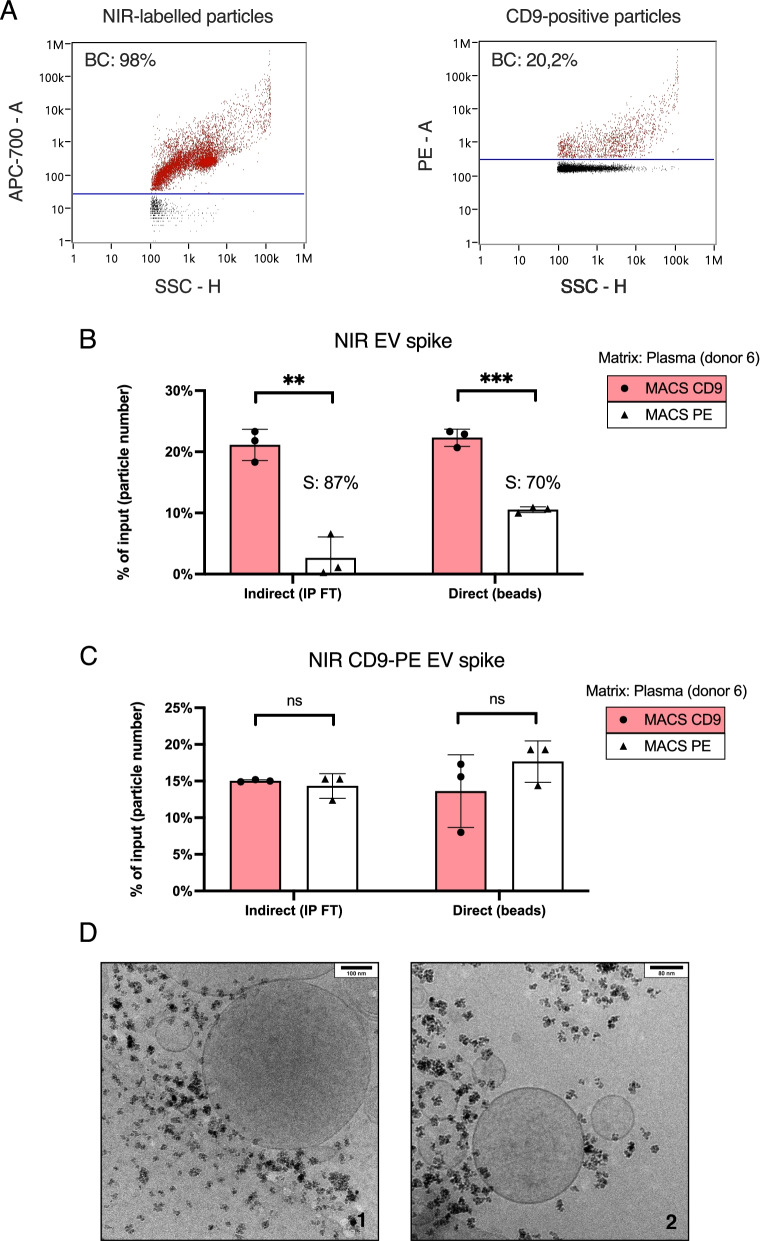
Intact EV subpopulations spiked in plasma can be completely recovered using antibody-conjugated beads. (**A**) Single-particle analysis of fluorescent 22RV1-NIR EVs by nFCM. The dot plot on the left shows that the majority of 22RV1 EVs incorporated the NIR fluorophore, whereas the one on the right evidences the CD9-positive subpopulation of these NIR-EVs, determined after staining with CD9-PE. Percentages of fluorescent particles were background-corrected (BC) with buffer (PBS). (**B**) IP of 22RV1-NIR EVs spiked in plasma (donor 6). Recovery (% of input) was appreciated indirectly, through the NIR signal of IP flow-throughs (FT) and directly, by measuring the fluorescence of NIR EVs captured on beads. 22RV1-NIR EVs were used to plot a calibration curve correlating particle numbers with their fluorescent signal, which allowed to present percentages of input based on the actual number of particles recovered vs. input particles. Specificity (S) was calculated for both readouts. (**C**) IP of 22RV1-NIR-CD9-PE-stained EVs spiked in plasma (donor 6). Recovery, plotted as % of input, was assessed by indirect and direct fluorescence readouts. The NIR signal of 22RV1-NIR-CD9-PE EVs was used to plot a calibration curve, correlating particle numbers with their fluorescent signal, which allowed to present percentages of input based on the actual number of particles recovered vs. input particles. Recovery with each bead source was equivalent, as suggested by both readouts. (**D**) Cryo-TEM images of EVs captured from plasma (1) and plasma spiked with HEK293 EVs (2), using triple-coated beads. Respective scale bars are shown on the top right corner of each image

**Fig. 3:**
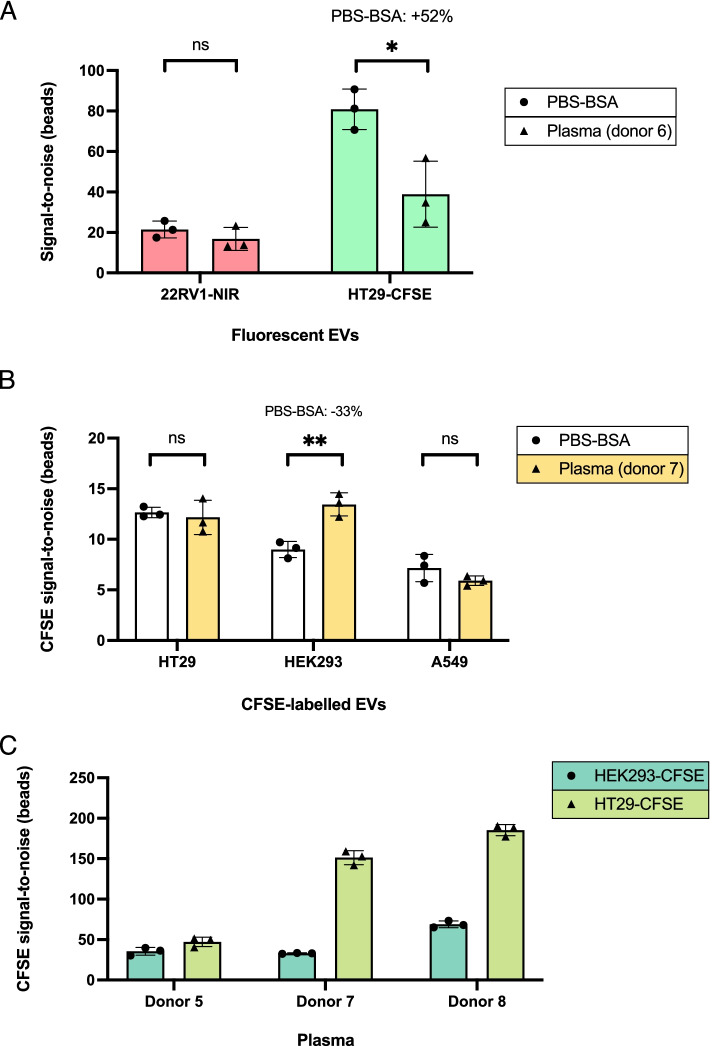
IP efficiency is dependent on EV surface properties, complex matrix components and interactions between both. (**A**) Fluorescence S/N ratios were assessed on triple-coated beads upon the IP of 22RV1-NIR and HT29-CFSE spiked in either PBS-BSA or plasma (donor 6). Average recovery (S/N) differences between the two matrices were reported in percentage. (**B**) CFSE-labelled EVs were spiked in plasma (donor 7) or in PBS-BSA and recovered using triple-coated beads. Average recovery (S/N) differences between the two matrices were reported in percentage. (**C**) CFSE-labelled HEK293 (blue) and HT29 EVs (orange) were spiked in plasma samples of 3 different donors and recovered using triple-coated beads. CFSE S/N on beads was directly proportional to the amount of captured spike

Subsequently, beads, EVs and the IP immune complex formed between them were visually examined by Cryo-TEM. An irregular bead structure could be discerned (Fig. 1B-1), contrasting with the circular shape of EVs and their well-defined membrane (Fig. 1B-2). IP complexes revealed that several beads can decorate the EV surface, which is likely dependent on the number of epitopes available for binding, amongst other factors (Fig. 1B-3).

Next, we evaluated the suitability of covalently-conjugated and streptavidin beads for EV IP in plasma, likely the most complex human biofluid. CFSE-labelled HEK293 EVs were spiked in healthy donor plasma (donor 6) and IP reactions conducted as aforementioned. Only direct bead readouts were plotted, since plasma emitted a great deal of background fluorescence in the CFSE channel, which compromised indirect readouts. Surprisingly, more fluorescent EV spike was recovered by negative control antibodies than with anti-CD9-coated or conjugated beads, on average (Fig. 1C). Such results would imply the lack of specificity of both bead surfaces in the plasma matrix, which was considered true for MACS-STV, as negative control beads were coated with a human isotype control antibody. Nevertheless, the negative control for the covalently-conjugated MACS beads was anti-CD61. Even though our spiked EVs did not express CD61, due to its abundance in plasma we reasoned that this platelet-related marker could be interacting with fluorescent EV spikes, causing their co-IP.

To confirm that the direct fluorescence readout accurately portrayed spike recovery, we extracted RNA from MACS-pulled down material (from Fig. 1C) and performed ddPCR for GAPDH and CA9, a stably transfected marker over-expressed in our HEK293 cells that becomes incorporated in their EVs, which is undetectable in healthy human plasma [63, 64]. The expression of both markers faithfully correlated with the previously measured fluorescent signal (Fig. 1C-MACS) and CA9 reads confirmed the specific recovery of our spike (Fig. 1D), indicating that fluorescence detected on beads stemmed from CFSE-labelled HEK293 EVs rather than from potential plasma-derived contaminants. Thus, ddPCR validated direct fluorescent measurements as reliable readouts of IP recovery.

To find trustworthy negative controls for assessing IP specificity from plasma, we re-tested the IP of CFSE-labelled HEK293 EVs spiked in plasma with MACS-CD9 against anti-phycoerythrin (PE) coated MACS beads (MACS-PE). PE is a commonly employed fluorophore produced by algae, which makes PE-coated beads an ideal negative control for IPs in human plasma. Spike recovery was now on average 4.5x higher with MACS-CD9 than with MACS-PE, conferring 78% of specificity to covalently-conjugated MACS beads in this experiment (Fig. 1E) and confirming our suspicion that CD61 was promoting spike co-IP.

Intrigued by this CD61-mediated capture of CD61-negative fluorescent EV spikes in plasma, we evaluated the recovery of CFSE-HEK293 EVs from the plasma of a different donor (donor 7), using MACS-CD9, CD61 and PE. Remarkably, CD61-mediated co-IP of CFSE spikes was not observed on a different plasma source as both negative controls displayed comparable fluorescence signals (Sup. Fig. 1C), indicating that this effect is dependent on biological variation.

In summary, antibody-coated MACS-STV specifically captured EVs only in a simple matrix, though they were markedly outperformed by antibody-conjugated MACS beads, which captured the totality of the CD9 subset in PBS-BSA and maintained a substantial degree of specificity, even in complex matrices. Therefore, we confirmed that the streptavidin-biotin surface chemistry is more prone to non-specific interactions in affinity-based EV isolation strategies. Importantly, both fluorescence measurement strategies and ddPCR proved to be valuable readouts that complemented and validated each other for precise quantifications of IP recovery.

### Whole EV subpopulations can be efficiently captured from plasma, while spike recovery is dependent on EV surface phenotypes and biological variation of complex matrices

Having selected covalently-conjugated MACS beads due to their superior performance, we aimed at optimizing and exploring their capability to capture specific EV subsets from plasma. When we attempted to read CFSE-stained spike inputs and IP flow-throughs in plasma, S/N ratios were too low to extract meaningful information. Upon light absorption, plasma emits plenty of blue/green autofluorescence, which ultimately masked CFSE signal. Because biomolecules absorb and emit almost no NIR light, fluorescent NIR probes are a promising tool for in vivo and ex vivo imaging [65–67]. For this reason, we generated endogenously-labelled NIR EVs by feeding 22RV1 cells with a NIR probe, which is internalized and stably latches on to lipidic membranes, even after EV secretion [60]. After SEC purification of 22RV1-NIR CCM, we detected 92% of NIR-fluorescent particles and observed a CD9 expression of 20% (Fig. 2A). To understand if the entirety of a single EV subpopulation could also be retrieved from complex matrices, not only NIR but also CD9-PE-labelled NIR EVs were spiked in plasma, followed by IP with anti-CD9 and anti-PE beads. As expected, NIR spikes delivered better S/N ratios in the plasma matrix, with respect to CFSE spikes, which allowed reliable input measurements and both direct and indirect IP readout reporting. In line with previous plasma experiments, we estimated 87 and 70% of specificity through indirect and direct readouts, respectively, during NIR spike IP (Fig. 2B). Moreover, both readouts evidenced a 20% recovery of NIR input, which exactly matched the proportion of CD9-positive 22RV1-NIR EVs, suggesting that the whole CD9 subpopulation of spiked EVs could be retrieved from plasma (Fig. 2B).

Interestingly, the recovery of CD9-PE-labelled NIR EV spike was comparable between CD9 and PE beads. The direct bead readout even evidenced a slightly higher mean of 18% for PE over 14% for CD9 (Fig. 2C), which could hint that CD9 epitopes may be less accessible to MACS-CD9 when anti-CD9-PE had already occupied them. These experiments confirmed the high efficacy of this IP approach for recovering distinct EV subsets from plasma, further highlighting its specificity and flexibility also by indirect capture.

IP complexes formed in plasma were monitored by Cryo-TEM, where an abundance of beads over EVs could be appreciated, while the size of captured EVs spanned over a wide range (Fig. 2D). Whether EVs were recovered from plasma (Fig. 2D-1) or from HEK293-spiked plasma (Fig. 2D-2), IP complexes greatly resembled the ones observed after IP in simple matrices (Fig. 1B), demonstrating that actual EV-like particles, with intact structure and function, could be efficiently retrieved from complex matrices.

Still, we reckoned that IP reactions would be more efficient in PBS-BSA than in plasma, due to the richness of the latter in biomolecules that can hinder affinity interactions. To assess that, we spiked 22RV1-NIR and HT29-CFSE in both matrices, conducted IP with triple-coated, anti-tetraspanin (CD9, CD63 and CD81) MACS beads and read their recovered fluorescence. On average, spike recovery was similar between buffer and plasma with 22RV1-NIR and 52% higher in buffer than in plasma with and HT29-CFSE (Fig. 3A). This observation suggested that depending on the identity of EV spikes, different interactions between EVs, matrix components and affinity reagents likely occur, affecting IP recovery.

To evaluate the impact of IP conditions on spike recovery, HT29-CFSE EVs were spiked in plasma from donor 6 (the same used in aforementioned experiments) and triple-coated MACS incubated for 10, 25 or 60 min. We confirmed a maximum average fluorescence signal at 60 min, whilst maintaining specificity (Sup. Fig. 2A). Moreover, the same HT29-CFSE spike was equally captured from donor 6 plasma increasingly diluted with PBS (Sup. Fig. 2B), showing that matrix dilution did not improve IP performance.

Having confirmed that IP conditions did not contribute to the variable recovery of different EV spikes, we further addressed this aspect by spiking CFSE-labelled EVs from three different cell lines (HT29, HEK293 and A549) in PBS-BSA and plasma from a different donor (donor 7), applying triple-coated MACS for IP. This time, fluorescent signals showed that HT29 EVs were equally recovered from both matrices. Similarly, the recovery of A549 EVs was not significantly different between the two matrices, while surprisingly, 33% more HEK293 spike was captured from plasma (Fig. 3B). In conclusion, such results demonstrate that the surface properties of distinct EV subsets can influence on how they are targeted and retrieved by affinity reagents, within a given matrix.

The complexity of plasma samples, exacerbated by wide inter-individual variation, is one of the major factors limiting clinical use of affinity-based assays. The disparity observed in HT29 EV recovery between PBS-BSA and plasma from donors 6 and 7 in two independent experiments (Fig. 3A, B), prompted us to estimate the real impact of biological variation on spike IP from complex matrices.

For this purpose, CFSE-labelled HEK293 and HT29 EVs were spiked into three different plasma sources and their recovery was assessed through direct IP fluorescence readouts. The recovery of HEK293 spike was similar between plasma samples of donors 5 and 7, although it doubled in donor 8 plasma. Plasma from donor 5 resulted in the lowest recovery of HT29 EVs, as this signal tripled in donor 7 and reached its maximum in donor 8 plasma (Fig. 3C). Intriguingly, the recovery of HEK293 EVs from plasma samples of donors 5 and 7 remained constant, while it tripled for HT29 EVs, further highlighting the weight of EV surface phenotypes in IP efficiency. Taken together, these results show how the affinity isolation of EV subpopulations depends both on their inherent surface characteristics, and on the composition of the matrix they are carried in.

### Multiple surface markers can be directly detected to quantify EV subpopulations captured from simple and complex matrices

**Fig. 4:**
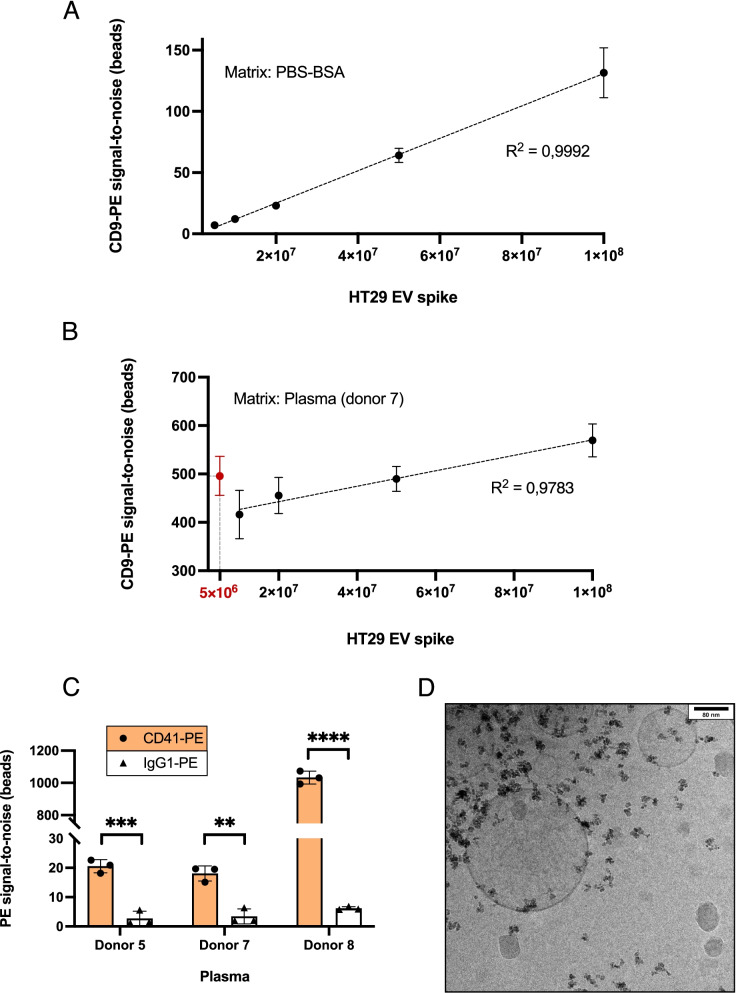
Direct EV subset detection and quantification on beads using fluorescently-tagged antibodies. (**A**) Increasing numbers of HT29 EVs captured from PBS-BSA with triple-coated beads were detected by staining IP complexes with CD9-PE. PE fluorescence signals highly correlated with the number of spiked and recovered EVs, evidenced by linear regression analysis (*R*^2^ = 0,9992). (**B**) Scalar amounts of HT29 EVs were spiked in “EV-depleted plasma” (donor 7) and recovered using triple-coated beads, followed by staining with CD9-PE. A linear trendline showed correlation between the amount of spike and S/N obtained with CD9-PE, from 1 × 10^8^ to 1 × 10^7^ EVs (*R*^2^ = 0,9783). An outlier mean S/N value at 5 × 10^6^ spiked EVs was presented in red. (**C**) Platelet-derived EVs were isolated from plasma samples of 3 different donors using anti-CD61 beads. Target subpopulations (CD41-PE) were detected against a negative control antibody (IgG1k-PE). (**D**) Cryo-TEM image of platelet-derived EVs recovered from plasma with anti-CD61 beads. Scale bars are shown on the top right corner of the image

Upon optimization and characterization of this IP approach employing fluorescently-labelled EV spike-in models, we attempted instead to stain captured EVs directly on beads, using fluorescently-tagged primary antibodies. With the goal of developing a strategy to quantitatively detect EV subpopulations retrieved from plasma, we initially set out to gauge the staining of bead-bound HT29 EVs with CD9-PE, directly after IP with triple-coated MACS in PBS-BSA. S/N ratios obtained on increasing EV numbers could be faithfully represented by simple linear regression (*R*^2^ = 0,9992), from 1 × 10^8^ down to 5 × 10^6^ EVs, which corresponded to a S/N of 7 (Fig. 4A). As such, this strategy revealed quite robust for EV detection and quantification in simple matrices. To understand its applicability in plasma, we attempted to first deplete endogenous plasma EVs with triple-coated MACS, then HT29 spikes were added to this “EV-depleted plasma” and IP was performed, using also triple-coated beads. Detection with CD9-PE displayed a linear trend from 1 × 10^8^ to 1 × 10^7^ HT29 EVs (*R*^2^ = 0,9783), however at the lowest spike amount (5 × 10^6^), an unexpected sharp increment in S/N ratios was noticed (Fig. 4B). Moreover, CD9-PE S/N ratios were substantially larger in plasma-derived bead samples (Fig. 4A, B), which suggested that either the pre-IP depletion step was incomplete, or that the majority of signal stemmed from nonspecific antibody binding.

To address the specificity of fluorescently-labelled primary antibody staining of plasma-derived material on beads, platelet-derived EVs were isolated from plasma samples of 3 independent donors, using MACS-CD61. Detection was done by targeting CD41, a platelet-related marker that forms a heterodimer with CD61 known as integrin αIIbβ3, present exclusively in the platelet lineage [68, 69]. A PE-labelled isotype-matched antibody was used as negative control. Specific CD41-PE signal was measured with different intensity across all three plasma samples, always significantly higher than respective negative controls, confirming that this bead-based sandwich immunoassay assay could specifically detect surface markers carried on EVs retrieved from plasma (Fig. 4C). Through Cryo-TEM we verified that, consistent with aforementioned images, MACS-CD61 clearly enabled the isolation of EV-like structures, suggesting that platelet-derived EVs could be efficiently captured from plasma (Fig. 4D).

**Fig. 5:**
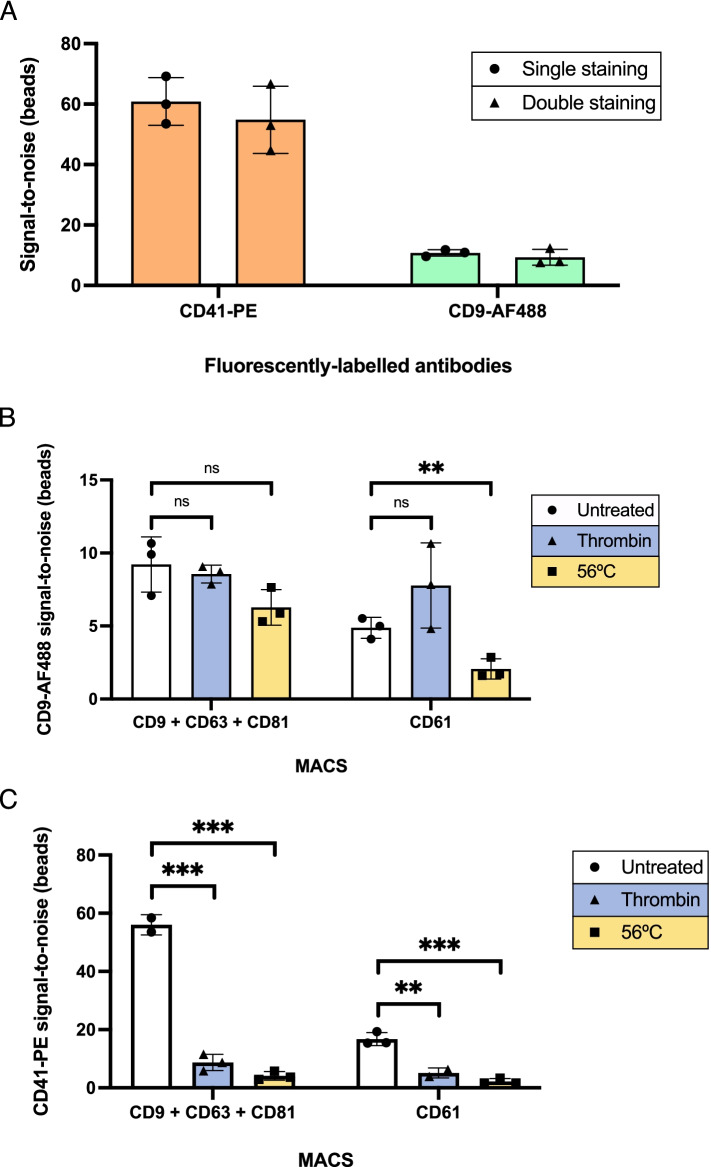
Double antibody staining of IP complexes allows for meaningful quantification of surface markers on plasma-recovered EVs. (**A**) Platelet EVs isolated from plasma with anti-CD61 beads were stained with CD41-PE and CD9-AF488 in single or double staining settings. Fluorescence signal for both markers was equivalent, despite the incubation with 1 or 2 antibodies simultaneously. (**B**) Triple-coated or anti-CD61 beads were incubated in untreated, thrombin treated or 56 °C heated plasma and double stained with CD41-PE and CD9-AF488. S/N ratios obtained with CD9-AF488 were plotted. A significant drop in fluorescent signal could be appreciated only upon 56 °C treatment, when CD61 beads were employed. (**C**) Measurements of CD41-PE, referring to the experiment described in (B). Substantial losses in CD41-PE signal were detected after staining IP complexes recovered with both beads, across treatments. Untreated and thrombin from triple-coated and CD61 beads, respectively, are shown as duplicates

Subsequently, we explored the possibility of simultaneously detecting two markers through double staining of platelet EVs, isolated from plasma with MACS-CD61. S/N ratios obtained after staining with anti-CD41-PE and anti-CD9-AF488 were comparable, regardless of their incubation being conducted in single or in combination (Fig. 5A), meaning that staining efficiency and accuracy is maintained as two markers are concomitantly detected.

Finally, we investigated if double staining could provide meaningful information in the analysis of EV subpopulations derived from complex samples, exploiting platelet-derived EVs as a paradigmatic example. To do so, 1 mL of plasma was first heated to 56 °C or treated with thrombin (2 U) for 8 min. Both procedures cause the precipitation of fibrinogen from plasma, noticeable by increased opacity or by the polymerization of an insoluble clot after 56 °C or thrombin treatment, respectively [70, 71]. For both treatments, insoluble fibrinogen was eliminated by centrifugation at 5000 g for 5 min and the resulting supernatant collected in a clean tube, to which either triple-coated MACS or MACS-CD61 were added. Since fibrinogen strongly interacts with the CD41/CD61 complex (also termed the fibrinogen receptor, required for clot formation) on platelets [72], we postulated that the effects of such treatments would mostly reflect on the detection of platelet markers, on platelet-derived EVs. To verify it, CD9-AF488 and CD41-PE double staining was conducted on recovered beads after incubation with treated and untreated plasma.

Fibrinogen-depleting treatments did not majorly impact CD9 detection on triple-coated MACS, however on MACS-CD61, a significant drop in fluorescent signal could be appreciated upon plasma pre-heating at 56 °C (Fig. 5B). On the other hand, sharp losses of CD41-PE signal were observed after treatments, on both triple-coated MACS and on MACS-CD61 (Fig. 5C). These results corroborated our hypothesis, as mostly platelet-related markers were indeed lost upon thrombin or 56 °C treatment, indicating that, not only fluorescent EV spike-ins but also endogenous plasma EVs were specifically captured from plasma samples, and that multiple EV subpopulations from complex samples can be simultaneously detected using this staining protocol. Of note, neither of the plasma pre-analytic treatments aforementioned resulted in increased overall EV recovery.

Taken together, we established that a simple incubation step with fluorescently-labelled antibodies on EV-carrying beads, recovered from simple or complex matrices, enables accurate detection and quantification of multiple surface markers expressed on EV immunoprecipitates.

### Different EV subpopulations carry distinct mRNA biomarkers that can be valuable for liquid biopsy-based early-stage NSCLC detection

As any bona fide enrichment strategy depends on its’ specificity, we ultimately sought to provide definitive evidence to validate the performance of our IP protocol. To do so, the mRNA content of different plasma EV subpopulations, recovered either by triple-coated MACS or MACS-CD61, was profiled using the nCounter platform. Moreover, to inquire about the utility of this strategy in a real liquid biopsy scenario, both EV subpopulations were isolated from the plasma of two different cohorts, each composed of 14 donors. The expression of 594 transcripts was measured using the nCounter Human Immunology v2 Panel. To avoid biased conjectures and guarantee the quality of gene expression reads, a dedicated bioinformatics pipeline was developed, including the internal standard nCounter QC checks, exploratory data analysis (EDA), low-count gene filtering steps, normalization and DE analysis.

**Fig. 6:**
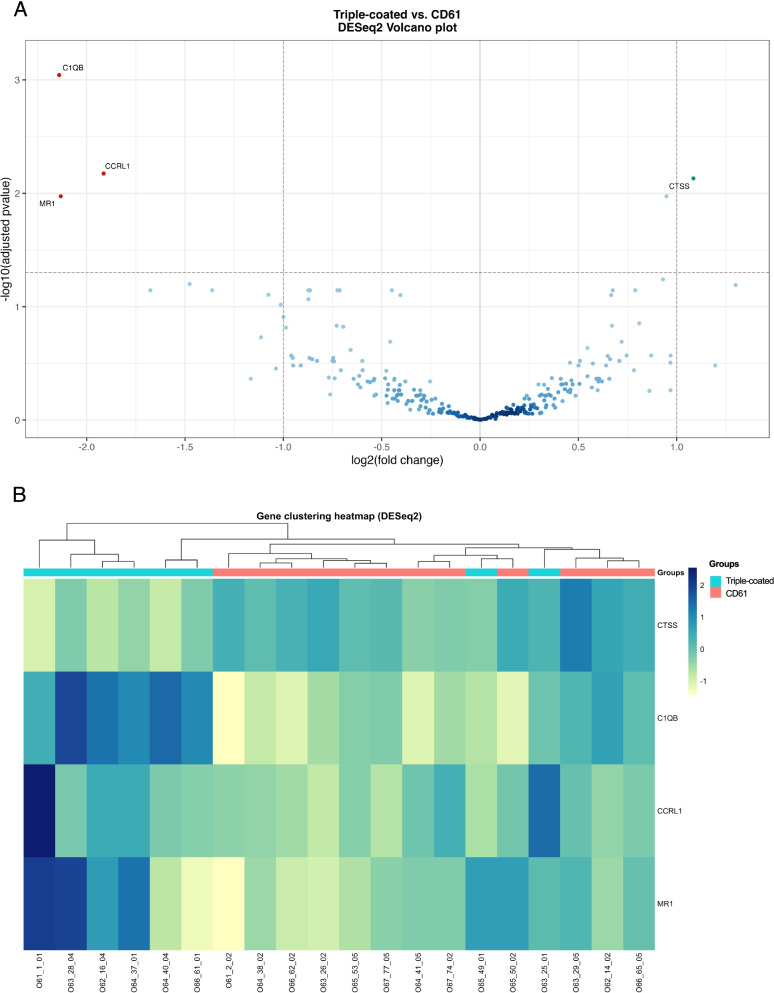
Differential expression (DE) analysis by DESeq2 between CD61+ and CD9, CD63 or CD81+ EV datasets on the healthy donor cohort. (**A**) Volcano plot showing DE genes between the two groups. Cut-offs were defined for adjusted *p-*values (0.05, Y axis) and log2 fold change (2, X axis). Upregulated and downregulated genes were depicted by green and red circles, respectively. (**B**) Supervised hierarchical clustering heatmap analysis based on the four DE genes discovered by DESeq2, samples and their respective group

Firstly, we questioned whether different EV subpopulations, derived from the same healthy donor samples, contained distinct mRNA profiles. During EDA on the comparison of healthy donor EVs obtained with triple-coated or CD61 beads, PCA revealed that samples seemed to slightly cluster by the number of unnormalized reads and group (Sup. Fig. 3A, B, C), but not by batch (Sup. Fig. 3D). However, after examining unnormalized counts per group, we concluded that CD61+ EVs displayed a significantly higher number when compared to CD9, CD63 or CD81+ EVs (*p* = 0.00053, Wilcoxon; Sup. Fig. 4A), indicating that the apparent clustering by group, defined in this case by IP target, did not truly occur, as it was driven by the number of mRNA counts. RLE plots demonstrated that optimal sample normalization could be achieved with DESeq2 (Sup. Fig. 3E, left). Normalized samples were visually inspected on a PCA plot, which evidenced the two most variable samples depicted on RLE plots (Sup. Fig. 3E, right). DESeq2 output four DE genes, one upregulated and three downregulated, when comparing MACS-CD61 against the triple-coated MACS dataset (Fig. 6A). Supervised hierarchical clustering analysis was performed using the four DE genes and presented on a heatmap (Fig. 6B). Altogether, our data supports that depending on the targeted surface markers, distinct EV subsets could be effectively isolated from healthy donor plasma.

**Fig. 7:**
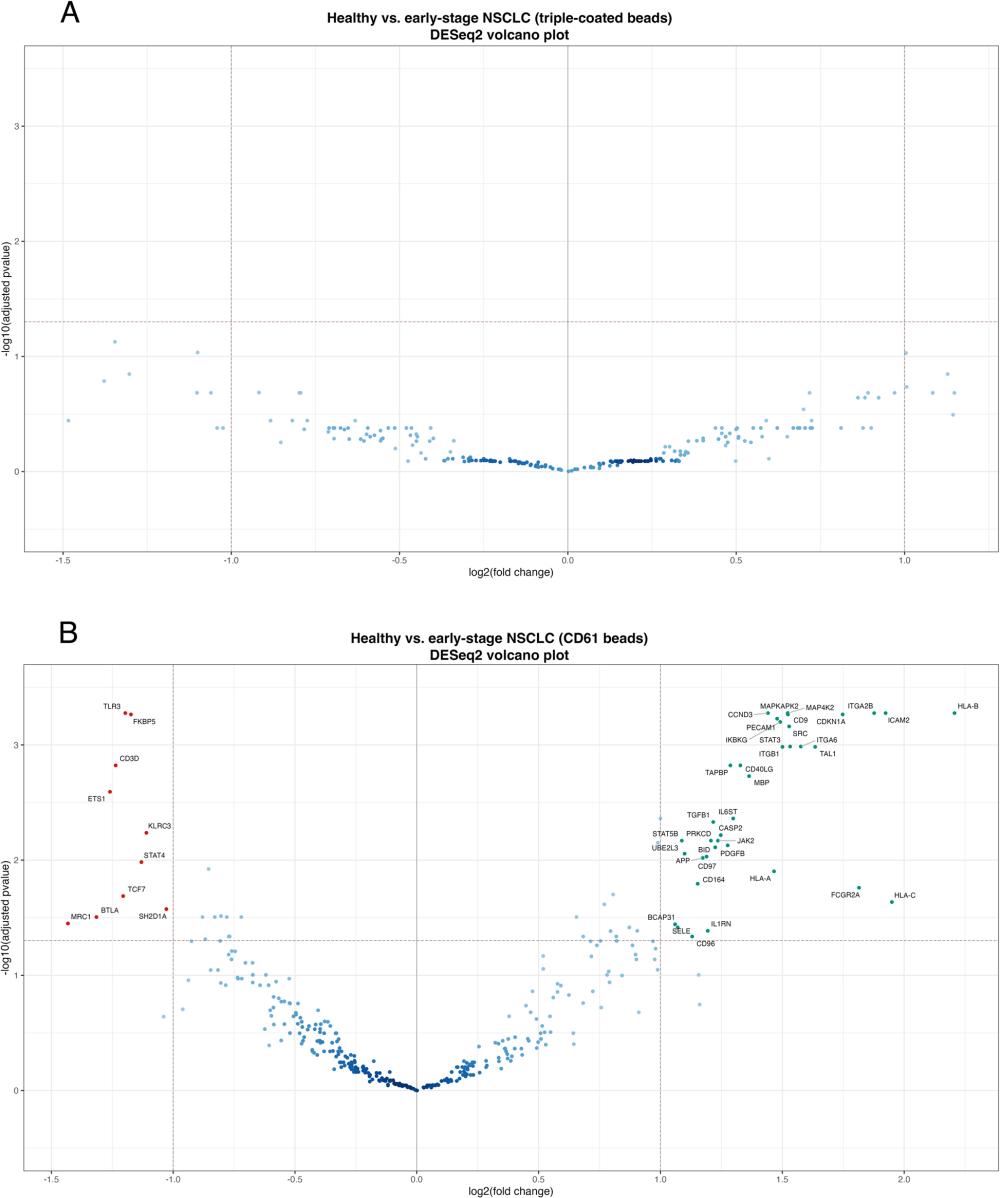
DESeq2 identified 47 DE genes between healthy and early-stage NSCLC cohorts, using platelet-derived EVs as biomarker source. (**A**) Volcano plot showing that no DE genes were found between the two cohorts, based on the triple-coated EV dataset. Cut-offs were defined for adjusted *p-*values (0.05, Y axis) and log2 fold change (2, X axis). (**B**) Volcano plot showing DE genes between the two cohorts, based on the platelet-derived EV dataset. Cut-offs were defined for adjusted *p-*values (0.05, Y axis) and log2 fold change (2, X axis). Upregulated and downregulated genes were depicted by green and red circles, respectively

To understand the potential clinical value of each EV subset as biomarker carrier, we confronted our healthy cohort against a prospective early-stage NSCLC cohort, applying the same pipeline. Surprisingly, no DE genes were found after comparing the EV mRNA profiles obtained from healthy and cancer samples, using triple-coated MACS (Fig. 7A). On the other hand, the platelet-derived EV dataset allowed to compare healthy and early-stage cancer cohorts. As previously observed during EDA, samples only seemed to somewhat cluster by the number of unnormalized gene counts, but not by group or batch (Sup Fig. 5A-D). Despite being marginally elevated, the average number of unnormalized counts was not significantly higher in the early-stage NSCLC cohort (*p* = 0.43, Wilcoxon; Sup. Fig. 4B). As before, DESeq2 alone optimally normalized all samples (Sup Fig. 5E left). Similarly, PCA plots of normalized counts evidenced a separation of the most variable samples (RLE plots, Sup Fig. 5E left) from the main sample cluster (Sup Fig. 5E right). DE analysis with DESeq2 found 47 DE genes, which were visualized on a volcano plot (Fig. 7B). These results suggest that the identified mRNA expression patterns displayed by CD61-positive EVs, may allow for distinction between healthy and early-stage cancer samples. In summary, our experimental data demonstrates that different EV subpopulations can indeed be captured by targeting different surface markers, which reflected on their mRNA profiles and disclosed how distinct EVs subsets may confer differential clinical values in relevant liquid biopsy settings. In this case, the identification of 47 putative biomarkers for blood-based early-stage NSCLC detection, revealed that platelet-derived EVs represent an appealing biomarker source that warrants extended studies.

## Discussion

In the present study, we thoroughly optimized a nanobead-based EV immunoaffinity capture strategy and demonstrate that distinct subpopulations can be efficiently isolated and selectively enriched, despite the complexity of IP matrices. Antibody-conjugated beads were chosen over streptavidin-coated ones as they allowed for superior EV recovery and purity with a simpler protocol, since bead coating steps were not required. Target EV subsets were equally recovered through indirect IP markers with similar efficiency, which showed the high flexibility enabled by antibody-conjugated beads, generally considered a hallmark of the streptavidin-biotin approach. Routine laboratory fluorescence readouts, validated through ddPCR and nFCM, delivered precise estimations of IP recovery. Cryo-TEM observation of bead-EV complexes formed in various IP matrices evidenced that actual membranous particles were decorated with beads, without noticeable signs of contaminating particles. Upon measuring fluorescent spike recovery between simple and complex matrices, we noticed that different EV surface properties could greatly impact IP efficiency. Moreover, we verified that not only the EV surface phenotype, but also biological sample composition can shape how EVs are retrieved through affinity capture. Altogether, the nano-scale trinity formed between EVs, matrix components and affinity reagents, comprising all their complex interactions, determines the success of IP reactions. We postulate that the same holds true under many other affinity-based EV enrichment conditions. The recovery of endogenous plasma EVs after pre-analytical treatments could be directly and accurately quantified using fluorescently-tagged primary antibodies, which supported the suitability of this straightforward detection method. Finally, triple-coated anti-tetraspanin (CD9, CD63 and CD81) beads and anti-CD61 beads were applied for EV IP, using merely 500 μL of human plasma obtained from 14 healthy donors and 14 early-stage NSCLC patients. Gene expression profiling of CD9, CD63 or CD81+ and CD61+ (platelet-derived) EVs revealed that each subset carried unique mRNA pools, emphasizing the selectivity of our IP approach, and the prevalent notion that EV subpopulations are highly heterogeneous. More importantly, we provided proof of principle for selective human EV enrichment strategies as valuable tools in a real-world clinical liquid biopsy context. Here, the mRNA pool carried within platelet-derived EVs better grasped differences between healthy and early-stage NSCLC samples, when compared to the tetraspanin-recovered EV subpopulation. We identified a relationship between 47 potential biomarkers for early tumour detection in platelet-derived EVs, while strikingly, none could be found in the tetraspanin-recovered subpopulation. It is important to consider that true identification of circulating plasma EVs is still extremely challenging. Currently available methodologies have yet to enable precise discrimination and profiling of both circulating nanoparticles and EV subpopulations. This topic certainly awaits further investigation, as it has been for years recognized in the field as a fundamental question. It is possible that the classical EV tetraspanin proteins (CD9, CD63 and CD81) might be loss, altered or obscured, as EVs diffuse through the vascular system, which would render them suboptimal IP targets.

Platelets have been extensively studied for biomarker discovery and detection, as they quickly respond to inflammation and can engulf numerous biomolecules sustaining pathophysiological states. Due to the uptake of tumour-shed material, platelets transform into tumour-educated platelets (TEPs) and actively start favouring cancer progression through processes extensively reviewed elsewhere [73, 74]. Platelet and TEP-derived EVs generate a positive feedback loop that amplifies platelet education and contributes to tumour growth. These EVs may also participate in metastatic processes as platelets do, however convincing evidence is still lacking [74–76]. Such ideas support the significance of platelet-derived EVs as valuable biomarker sources, which could actually be more enriched in disease-specific analytes than the platelets generating them.

The endpoint goal of our study lied in assembling a selective EV isolation approach, tailored to comply with ideal routine procedures required in clinical liquid biopsy environments. Realistically, it is extremely challenging to introduce new techniques into the clinic, as emphasized by Ignatiadis et al., who elegantly defined a roadmap for the translation of novel liquid biopsy assays into clinical practice [77]. Accordingly, we demonstrated the analytical validity and set off the clinical validation of our method, which simply required 500 μL of minimally processed PFP, while IP reactions were completed within 1 h. The superparamagnetic properties of beads can be leveraged for scalability and automation. Immunostaining conducted directly on the bead-EV complex latched inside magnetized columns, resulted in straightforward and reliable surface protein quantification, merely adding one extra hour to the protocol. It is important to stress that the detachment of immunocomplexes was not required, as MACS beads did not interfere with fluorescence measurements.

A similar bead-based MACS immunostaining protocol to profile EV surface markers has been developed [14]. Despite its convincing performance on the analysis of purified EVs, it cannot be directly applied in plasma samples. Moreover, this methodology relies on flow cytometry, which is not always straightforward, as instruments require precise optimization and frequent maintenance. Additional limitations stem from the size detection limit of conventional cytometers, which dictates that large size beads (4,8 μm) must be used for EV immobilization before fluorescence measurements, rendering the assay semi-quantitative. Also, it is likely that the large MACSPlex beads employed in this assay are easily outperformed by their smaller MACS beads (50 nm) counterpart [56], which were used in the present study. Our bulk fluorescence measurements obtained with a common plate reader provided the same kind of semi-quantitative information. We opted for direct (bead-EV complex) instead of indirect (IP flowthrough) IP readouts, as the former was conceptually more solid and better represented true EV recovery. Also, it could be applied regardless of the IP matrix or fluorescence of spiked EVs, and enabled in-column staining of the bead-EV complex.

One limitation in our experiments was the calculation of specificity. IP recovery always increased with the amount of spike when using specific antibodies against EV markers, while with negative control antibodies, it remained relatively stable. Logically, the ratio between the fluorescent signal measured in negative controls and targets becomes smaller as the number of EV input increases and consequently, specificity will also indirectly increase. In other words, the higher is the EV input, the higher we can expect specificity to be. We tried to minimize this effect by maintaining the number of spiked EVs constant across experiments. Moreover, since fluorescence does not vary linearly with quantity, this effect should ideally be accounted for. Therefore, we propose a less biased way to calculate specificity, based on the experiments depicted in Fig. 2, where calibration curves were plotted for the fluorescence of NIR spikes. In this way, we could convert NIR S/N ratios into numbers of NIR EVs, and were able to directly compare the number of particles recovered in both target and negative control beads, with respect to the initial particle number. However, this was only possible because the NIR dye was fed to 22RV1 cells, which released a population of nearly 100% endogenously-stained EVs. Since exogenous CFSE labelling was conducted on previously purified EV samples, the protocol required dye removal steps, which may still leave traces of free dye that can skew fluorescence calibration curves. Thus, this approach was avoided when reporting data obtained using CFSE-stained EV spikes.

Instead, antibodies in excess could be efficiently washed off after in-column staining of the bead-EV complex, for which we determined a lowest limit of detection (LOD) in the order of 10^6^ HT29 EVs upon anti-CD9-PE staining. Since PE is one of the brightest fluorophores available, it is unlikely that the LOD can be further extended by simply employing fluorescently-tagged primary antibodies. We want to underline that the LOD of this assay is surely influenced by the EV source and amount of target marker displayed, thus it should be experimentally confirmed in each particular setting. Spike detection after IP from plasma was accurate down to 10^7^ EVs. Lower spike amounts resulted in unrealistic S/N ratios, indicating that in this scenario, co-precipitated plasma material impeded specific antibody staining. Incomplete upstream depletion of plasma EVs might be responsible for this phenomenon, while EV-associated proteins might also circulate as individual soluble markers in plasma, which could limit the detection of low abundance EV targets through single primary antibody staining. Smaller affinity reagents such as nanobodies could potentially improve detection of nanostructures retrieved from plasma. Notwithstanding, the realistic availability of patient samples reflected on the need to employ low volumes of plasma samples. Minute amounts of biological material recovered from such samples were not sufficient for evaluation using orthogonal methods. Accurate detection of low-abundance co-precipitated contaminants through fluorescence-based measurements was also hampered. Still, a previously published study supported our conclusions, while addressing non-specific contaminant co-isolation by MACS beads. The authors compared the performance of several different affinity-based strategies for EV isolation from human serum [78]. According to their results, serum-derived EVs purified with MACS beads resulted in the highest detection of CD9, CD63 and CD81, while displaying the lowest degree of contaminant co-isolation, which included albumin and the apolipoproteins ApoB and ApoA1, among others [78].

Intriguingly, while experimenting with plasma from donor 6, we realized that anti-CD61 beads recovered more HEK293 spike than anti-CD9 beads. Our cell-derived EVs did not express CD61, suggesting that CD61-positive material captured from plasma mediated the co-IP of fluorescent spikes. We confirmed this by ddPCR, however the same effect could not be reproduced across plasma donors, hinting that potential matrix-dependant CD61-mediated EV co-IP interactions can only occur under certain circumstances. Integrins such as CD61 are fundamental regulators of cell communication, forming adhesive complexes that mediate extracellular interactions. They are usually found at the EV surface [23, 79], therefore it is conceivable that plasma-derived EVs, together with their associated proteins, can sustain the co-IP of other EV subpopulations. These events are related with an emerging concept in the EV field, named the EV protein corona, which comprises proteins that interact and associate at the surface of EVs [58, 80–82]. The dynamic nature of the EV protein corona and our limited understanding of its implications make it difficult to propose regulators or mediators of these interactions. Still, our observation of spike co-IP through CD61 and the drop in platelet markers after plasma treatments to eliminate insoluble fibrinogen, suggest that integrins, together with extracellular matrix proteins, such as CD61/CD41 (or integrin αIIbβ3) and fibrinogen, are surely potential candidates that will help in elucidating the extent of the plasma EV surface interactome. It is important to note that despite being an established cluster of differentiation of the platelet lineage, CD61 contributes to many additional functions and has been extensively implicated in cancer-promoting events, although further investigation is warranted before patients can benefit from CD61-targeted therapies [83].

The data depicted in Fig. 3C revealed to be quite surprising, especially when comparing the signal of both CFSE-stained spikes retrieved from the plasma of donors 5 and 7. The recovery of HT29 EVs from donor 7 plasma was 3-fold higher, however the same was not true for HEK293 EVs. Thus, we concluded that our HT29 spike selectively interacted with certain matrix components present in the plasma of donors 5 and 7, which either hampered or boosted its recovery (as observed in Fig. 3B for HEK293), respectively, using triple-coated beads. As the recovery of HEK293 spike from both plasma matrices was equal, we can state that the specific HT29 EV surface marker profile was responsible for this effect. It would be crucial to conduct systematic studies aimed at unveiling key surface regulators that mediate interactions between EVs and plasma matrix components, in order to improve and take full advantage of selective affinity-based EV isolation procedures. Notwithstanding, we also demonstrated that plasma obtained from each single individual can differentially impact the success of EV IP. Overall, this can be attributed to the wide variation in biological sample composition. Ultimately, we want to underline that both target EVs and the matrix they are transported in are critical factors to account for.

We conducted plasma pre-analytical treatments with two fundamental goals in mind: primarily, not only to prove the specificity of the IP technique but also of the bead-based sandwich immunoassay using fluorescently-labelled primary antibodies. Secondarily, to understand if simplification of the plasma matrix would increase endogenous plasma EV recovery, which in this case did not occur. In line with this finding, diluting the plasma matrix and its components in PBS did not result in increased spike recovery (Sup. Fig. 2B). However, due to our limited experiments, it is difficult to draw strong conclusions on this frontier and to claim whether or not pre-IP plasma processing would overall improve recovery. It is possible that thoroughly optimized pre-analytical parameters, from blood collection to EV IP, may benefit the affinity capture of at least some specific plasma-derived EVs subpopulations. Nonetheless, biological variability is an extremely difficult factor to control, hence it can be challenging to apply pre-analytical protocols that equally fit all patient samples. Further research could shed light on the fundamental plasma components hampering EV IP, contributing to minimize or standardize pre-analytical biases. Despite appraising pre-analytical challenges and the impact of complex matrix effects on endpoint biomarker detection, we uphold that our method was still robust enough to extract clinically relevant information from cancer patient EV samples, using as little as 500 μL of plasma.

The 47 DE genes identified after profiling platelet-derived EVs from the healthy and early-stage NSCLC pilot cohorts, displayed distinct expression patterns among samples. We found pronounced cancer-indicative profiles in stage II/III patient samples, which were reflected to a certain extent by some stage I NSCLC tumours, confirming that signatures of very early disease onset could be identified (unpublished observations). It is possible that not all patients might have had a detectable disease burden at early cancer development stages or instead, tumours could have progressed during the time elapsed post-prospective blood collection, until medical diagnosis. It will be critical to exclude the influence of pre-analytical and analytical processes on EV expression profiles to assure that true biological variation is observed. Due to the small size of our clinical cohorts, we took a conservative approach in this proof of principle study by simply interpreting these hints as remote indications, which will be further addressed in an ongoing, extended clinical prospective cohort study. Prospective studies aiming to pinpoint the time frames at which diseased plasma-derived EV profiles become detectable in different individuals, while closely monitoring tumour development and progression would be extremely valuable. Still, for broad clinical application of this and other novel liquid biopsy strategies, it is important to ensure that pre-analytical factors do not hamper early disease onset detection and that cancer-indicating signatures are not extensively shared with other pathologies.

## Conclusions

Lastly, we hope to encourage more research aimed at dissecting the complex interactions between EVs, matrix and affinity reagents. EVs are pivotal biomarker vaults that can be found across easily obtainable biofluids. However, identifying and retrieving the most relevant circulating EV subpopulations remains a central challenge in the field. As established by many exceptional reports during the last two decades, liquid biopsies hold the key for next-generation diagnostics and precision medicine. Ground-breaking research towards clinical implementation will propel the widespread dissemination of liquid biopsy-based tools, which will help to routinely guide medical doctors. Scalable and automatable procedures are essential to devise truly translational solutions, which meet the standards and expectations of clinical units.

## Supplementary Information

**Additional file 1: Supplementary Figure 1**. (**A**) Representative nFCM dot plot, showing a single-particle quantification of HEK293 EVs after CFSE staining. As determined after background correction (BC), nearly 88% of EVs incorporated the dye. (**B**) CD9 expression was determined by labelling HEK293 EVs with CD9-PE. Five independent experiments were plotted, resulting in a mean value of 42%. (**C**) HEK293-CFSE were spiked in plasma (donor 7) and IP conducted with triple-coated, anti-CD61 and anti-PE beads. Experiment performed in duplicate.

**Additional file 2: Supplementary Figure 2.** (**A**) Triple-coated or anti-PE beads were incubated for 10, 25 or 60 min in plasma (donor 6) spiked with HT29-CFSE EVs. Fluorescence signals were read on beads and specificity (S) determined. Highest average S/N ratios were obtained at 60min. Experiment performed in duplicate. (**B**) HT29-CFSE EVs were spiked in plasma (donor 6) diluted 1:2, 1:5, 1:10 or 1:20 with PBS, whilst maintaining a constant volume for IP with triple-coated beads. S/N ratios indicated that a similar recovery was achieved across dilutions. Experiment performed in duplicate.

**Additional file 3: Supplementary Figure 3.** mRNA profiles of CD61+ and CD9, CD63 or CD81+ EVs obtained from healthy donor plasma: exploratory data analysis and normalization. (A to D) Principal component analysis (PCA) was performed on unnormalized gene counts for exploratory data analysis. Principal components PC1 and PC2 were plotted on the X and Y axis, respectively. Different variables were evidenced: (**A**) Gene counts excluding internally-defined nCounter control genes, (**B**) Total read count per sample, (**C**) Sample groups, which in this case represented the antibodies immobilized on IP beads. Triple-coated (CD9, CD63 and CD81) or CD61-coated beads, (**D**) Due to a maximum number of 12 slots per nCounter experiment, samples were processed in different batches. Each batch represents one individual nCounter run. (E) Relative log expression (RLE) plots for visualization of the normalization performance with DESeq2 (left). Normalized count data was projected on PC1 and PC2 after PCA (right). Triple-coated and CD61 samples were represented in green and orange, respectively.

**Additional file 4: Supplementary Figure 4.** (**A**) Box plot evidencing raw counts obtained from healthy donor EV samples using both triple-coated or CD61 beads. A statistically significant difference between groups could be appreciated (*p-value* = 0.00053). (**B**) Box plot evidencing raw counts obtained from healthy donor and patient EV samples using CD61 beads. No statistically significant difference could be found between groups (*p-value* = 0.43).

**Additional file 5: Supplementary Figure 5.** EDA and normalization of gene expression data comparing two platelet-derived EV datasets obtained from healthy donors and early-stage NSCLC patients. (A to D) Principal component analysis (PCA) was performed on unnormalized gene counts for exploratory data analysis. Principal components PC1 and PC2 were plotted on the X and Y axis, respectively. Different variables are evidenced:(**A**) Gene counts excluding internally-defined nCounter control genes, (**B**) Total read count per sample, (**C**) Sample groups representing the two cohorts compared, healthy donors vs. early-stage NSCLC, (**D**) Due to a maximum number of 12 slots per nCounter experiment, samples were processed in different batches. Each batch represents one individual nCounter run. (E) Relative log expression (RLE) plots for visualization of the normalization performance with DESeq2 (left). Normalized count data was projected on PC1 and PC2 after PCA (right). Healthy donor and early-stage NSCLC patient samples were represented in green and orange, respectively.

**Additional file 6: Supplementary Table 1**. Quantification of bead input and EV spikes used across IP experiments. We assured that beads always exceeded the number of EVs, but also avoided an overabundance of the former.

## Data Availability

The datasets used and/or analysed during the current study are available from the corresponding author on reasonable request.

## Abbreviations

EVs: Extracellular vesicles
UC: Ultracentrifugation
SEC: Size-exclusion chromatography
CCM: Cell conditioned medium
IP: Immunoprecipitation
PBS: Phosphate-buffered saline
NSCLC: Non-Small Cell Lung Cancer
PRP: Platelet-rich plasma
PFP: Platelet-free plasma
nFCM: Nano flow cytometry
NIR: Near infra-red
SSC: Side scatter
S/N: Signal-to-noise
Cryo-TEM: Cryogenic transmission electron microscopy
DE: Differential expression
PCA: Principal component analysis
MDS: Multidimensional scaling
NC: Negative control
RLE: Relative log expression
MACS-STV: STREPTAVIDIN-conjugated beads
MACS: Covalently-conjugated beads
MACS-CD61: Anti-CD61-conjugated beads
MACS-PE: Anti-phycoerythrin (PE) coated MACS beads
EDA: Exploratory data analysis
TEPs: Tumour-educated platelets
LOD: Limit of detection
