# Supplementary figures and images for: Selective isolation of extracellular vesicles from minimally processed human plasma as a translational strategy for liquid biopsies
Source: Biomark Res. 2022 Aug 7;10:57. doi: 10.1186/s40364-022-00404-1 (PMC9357340; doi:10.1186/s40364-022-00404-1)

A

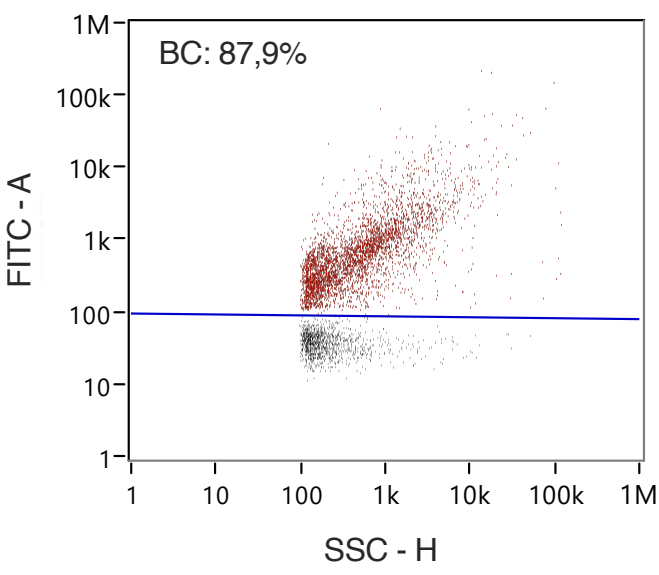

B

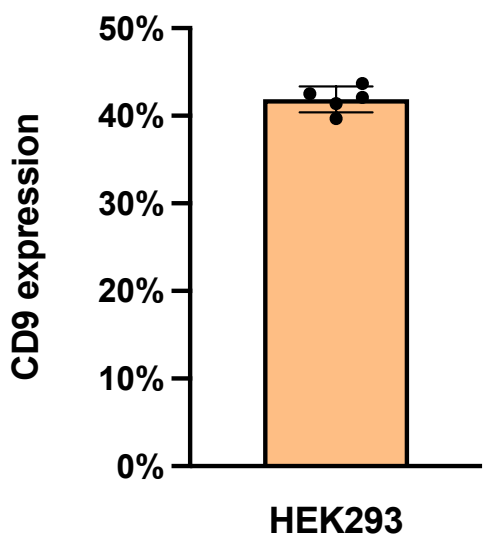

C

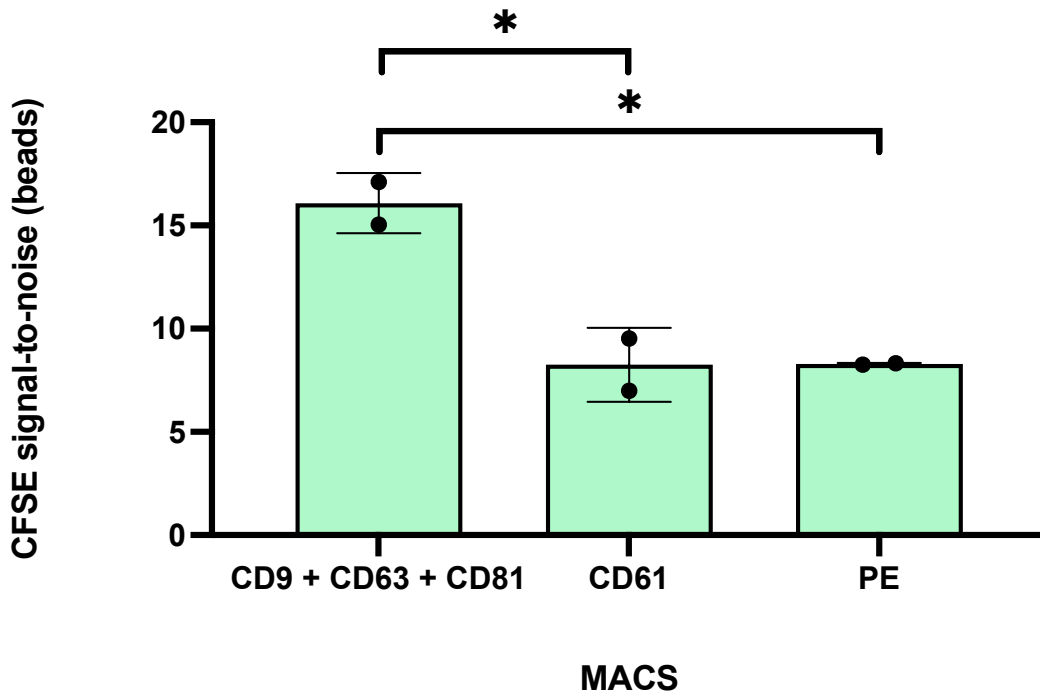

Supplement: Supplementary file 1 — Additional file 1: Supplementary Figure 1. (A) Representative nFCM dot plot, showing a single-particle quantification of HEK293 EVs after CFSE staining. As determined after background correction (BC), nearly 88% of EVs incorporated the dye. (B) CD9 expression was determined by labelling HEK293 EVs with CD9-PE. Five independent experiments were plotted, resulting in a mean value of 42%. (C) HEK293-CFSE were spiked in plasma (donor 7) and IP conducted with triple-coated, anti-CD61 and anti-PE beads. Experiment performed in duplicate. [file 40364_2022_404_MOESM1_ESM.pdf]

A

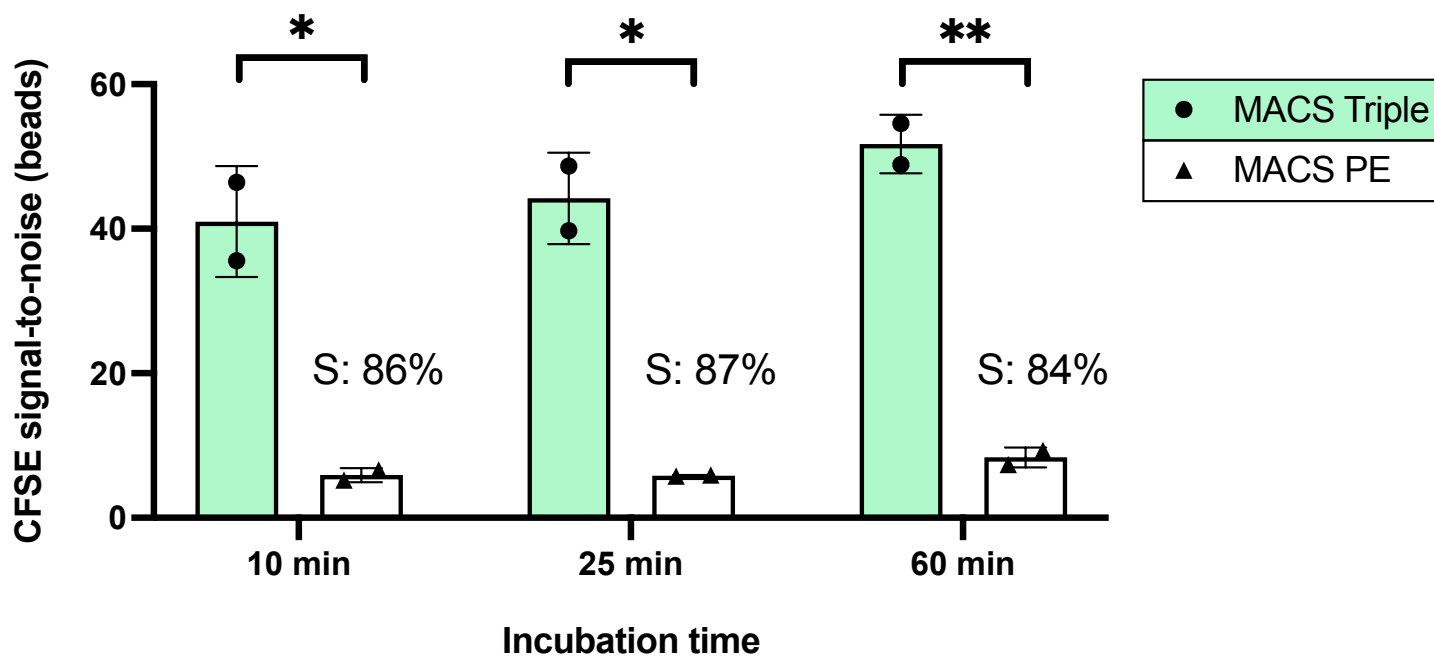

B

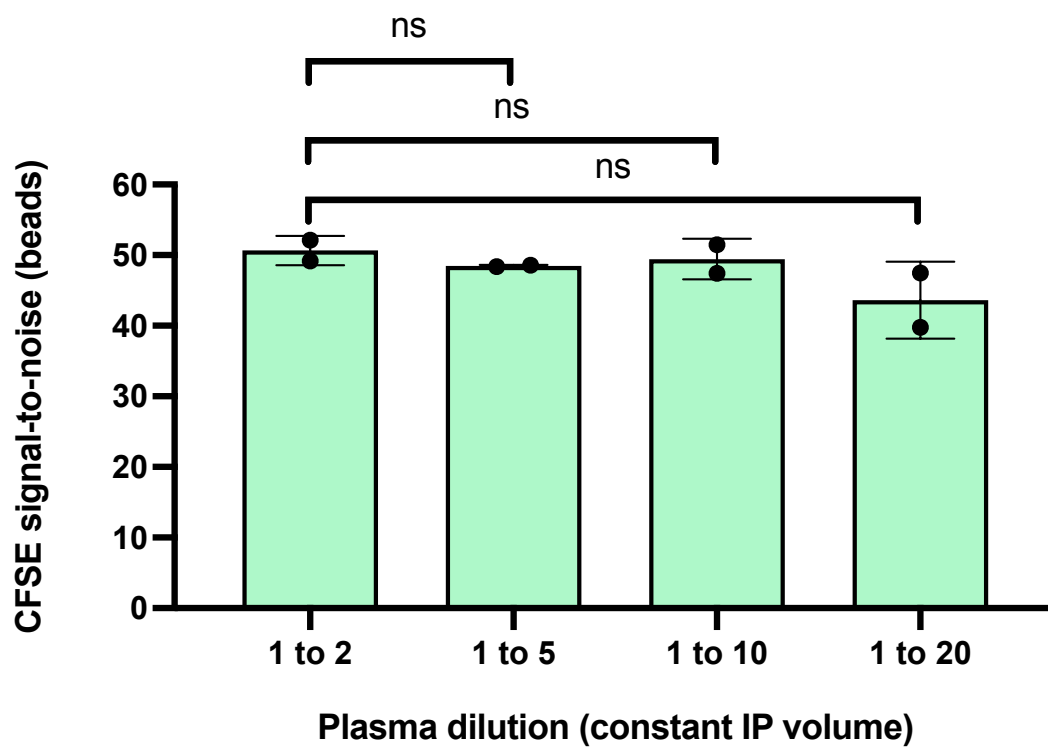

Supplement: Supplementary file 2 — Additional file 2: Supplementary Figure 2. (A) Triple-coated or anti-PE beads were incubated for 10, 25 or 60 min in plasma (donor 6) spiked with HT29-CFSE EVs. Fluorescence signals were read on beads and specificity (S) determined. Highest average S/N ratios were obtained at 60min. Experiment performed in duplicate. (B) HT29-CFSE EVs were spiked in plasma (donor 6) diluted 1:2, 1:5, 1:10 or 1:20 with PBS, whilst maintaining a constant volume for IP with triple-coated beads. S/N ratios indicated that a similar recovery was achieved across dilutions. Experiment performed in duplicate. [file 40364_2022_404_MOESM2_ESM.pdf]

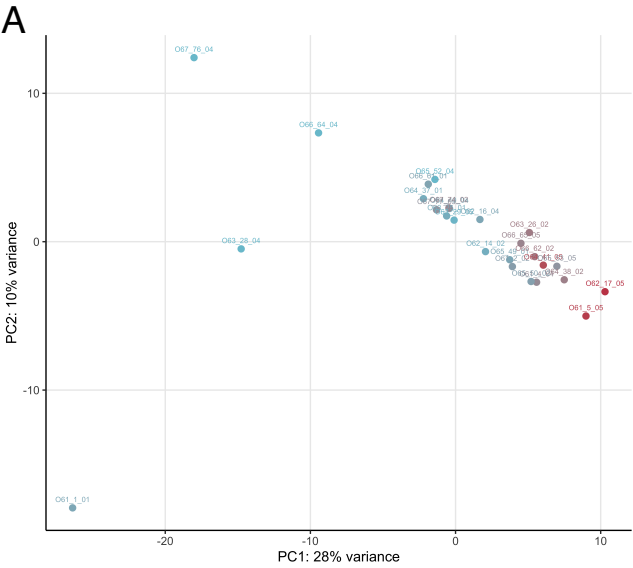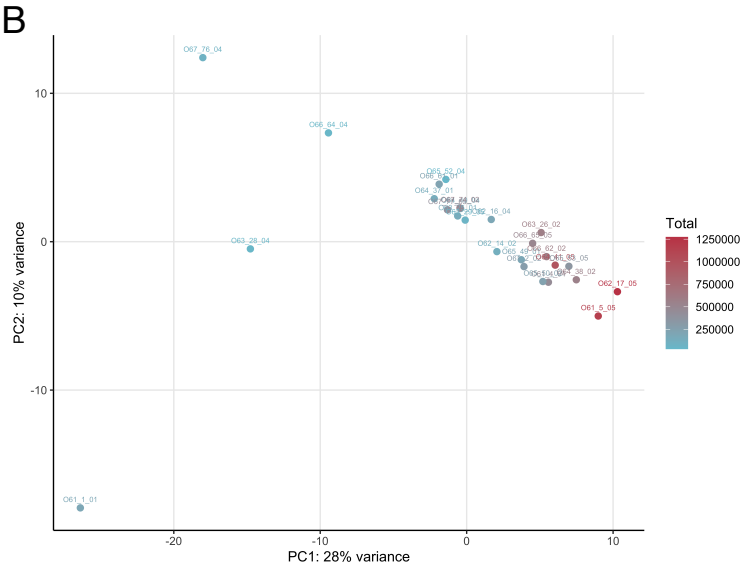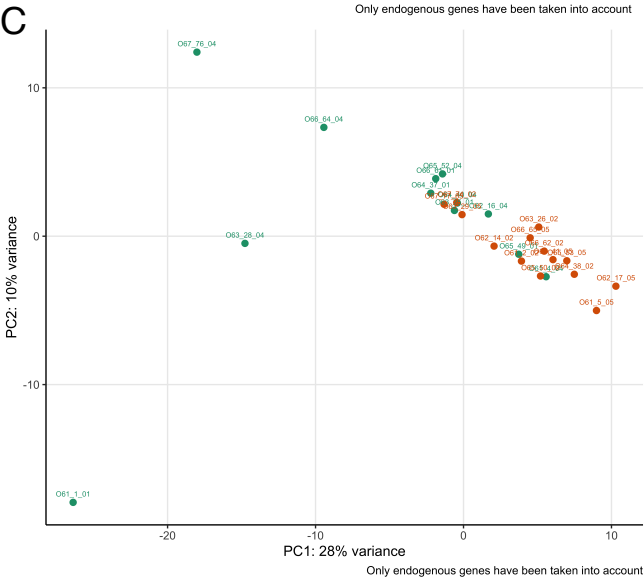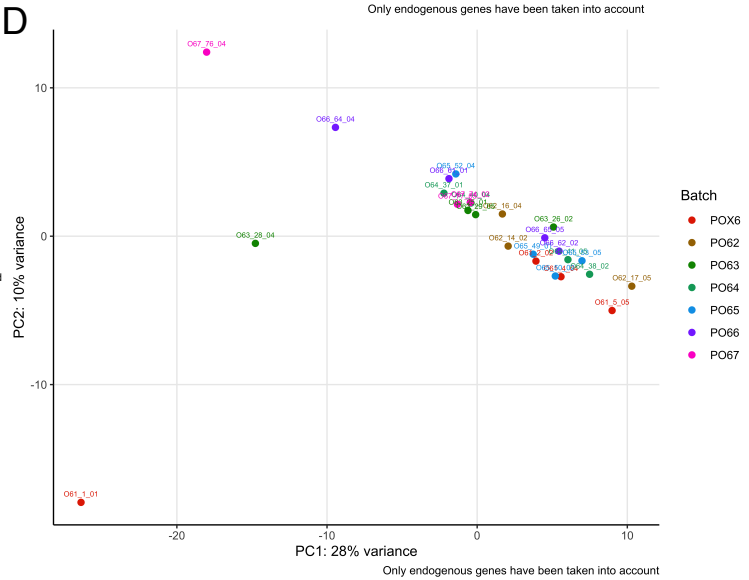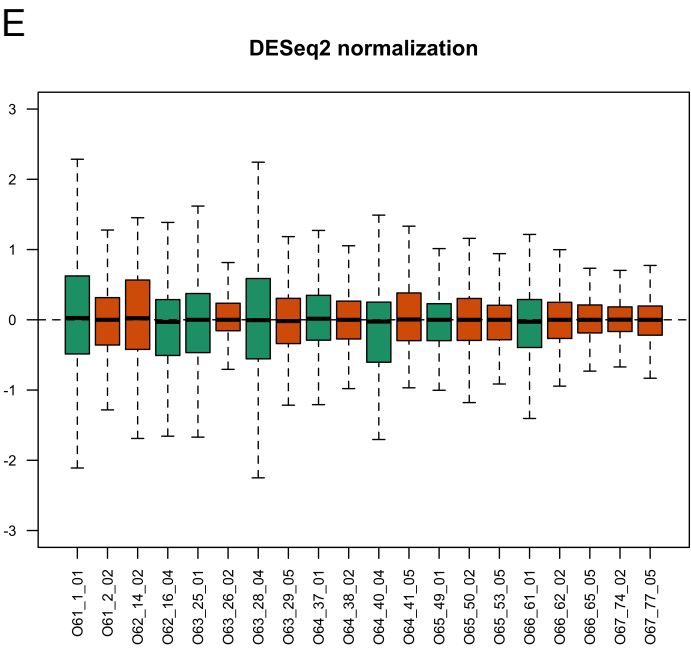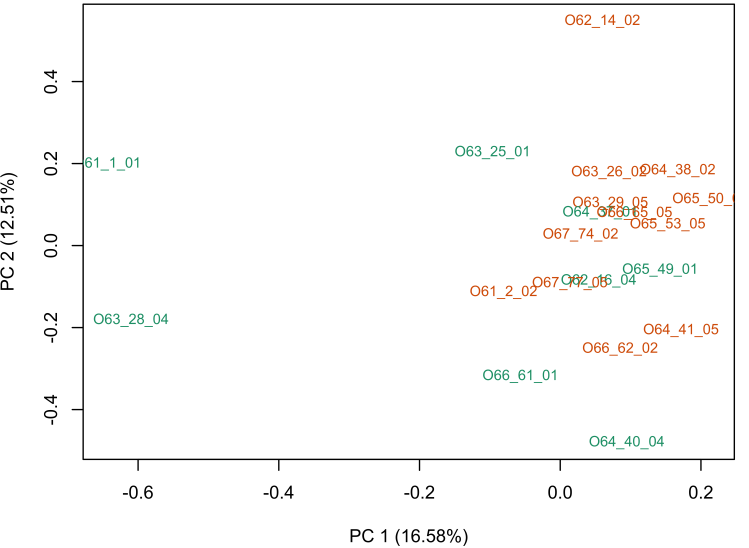

Supplement: Supplementary file 3 — Additional file 3: Supplementary Figure 3. mRNA profiles of CD61+ and CD9, CD63 or CD81+ EVs obtained from healthy donor plasma: exploratory data analysis and normalization. (A to D) Principal component analysis (PCA) was performed on unnormalized gene counts for exploratory data analysis. Principal components PC1 and PC2 were plotted on the X and Y axis, respectively. Different variables were evidenced: (A) Gene counts excluding internally-defined nCounter control genes, (B) Total read count per sample, (C) Sample groups, which in this case represented the antibodies immobilized on IP beads. Triple-coated (CD9, CD63 and CD81) or CD61-coated beads, (D) Due to a maximum number of 12 slots per nCounter experiment, samples were processed in different batches. Each batch represents one individual nCounter run. (E) Relative log expression (RLE) plots for visualization of the normalization performance with DESeq2 (left). Normalized count data was projected on PC1 and PC2 after PCA (right). Triple-coated and CD61 samples were represented in green and orange, respectively. [file 40364_2022_404_MOESM3_ESM.pdf]

A

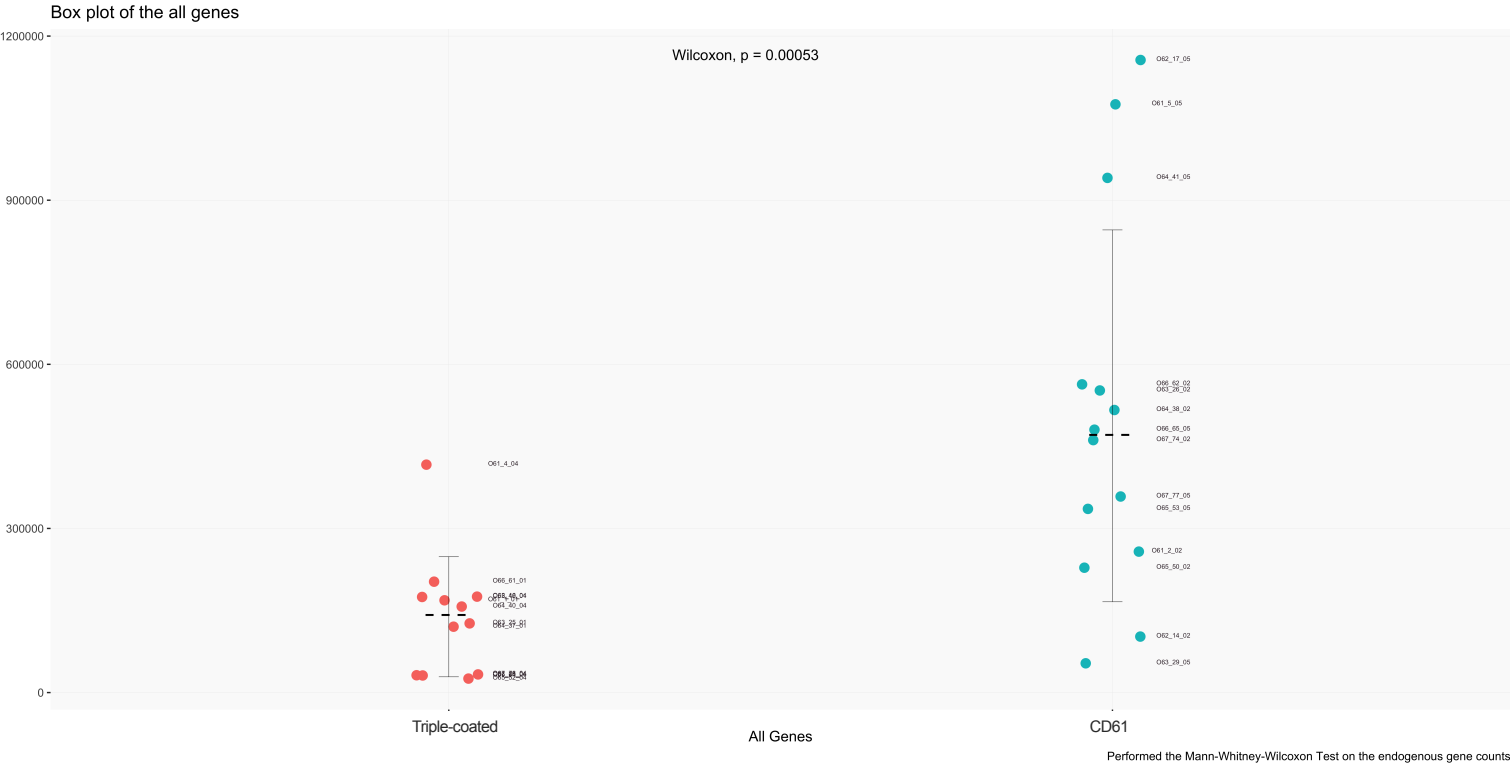

B

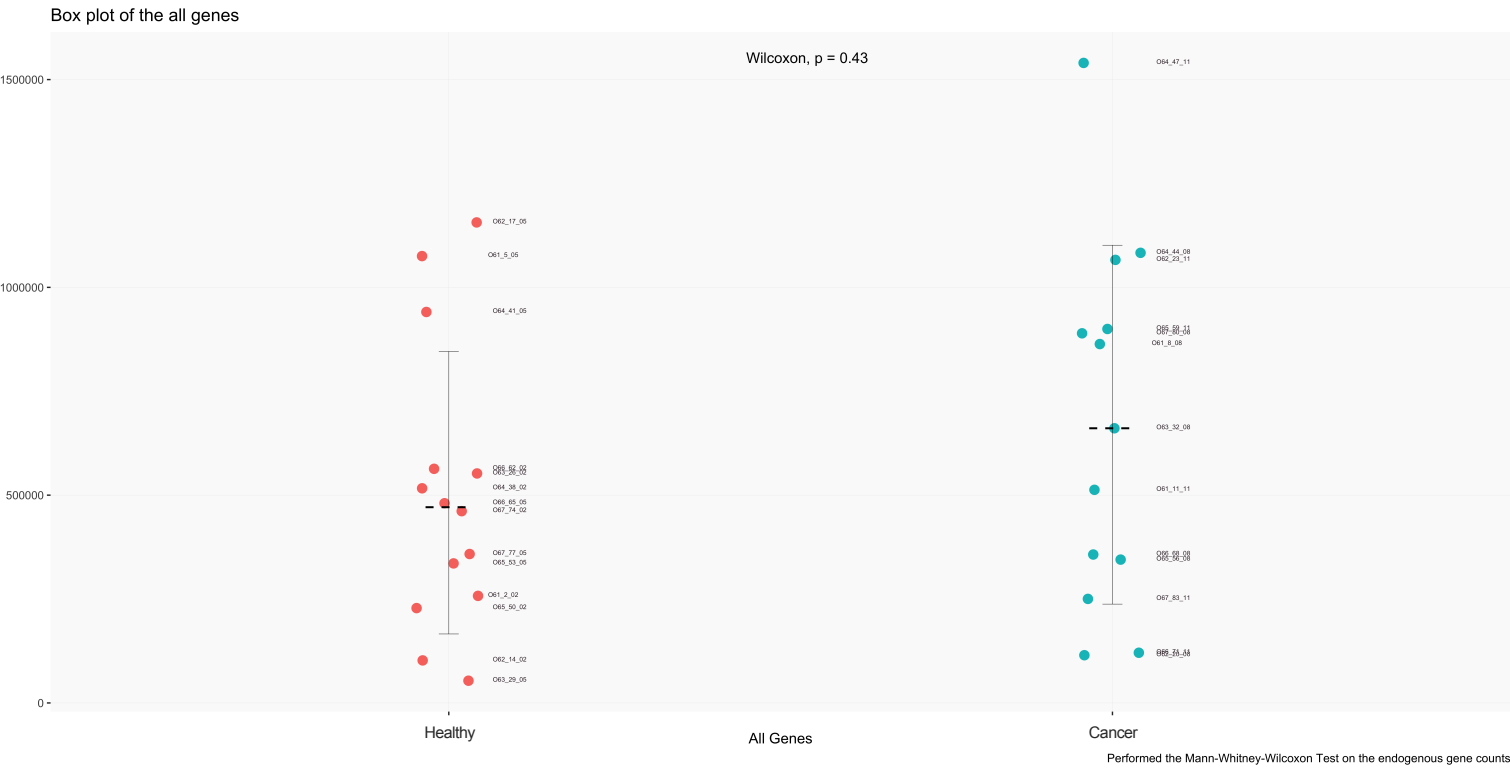

Supplement: Supplementary file 4 — Additional file 4: Supplementary Figure 4. (A) Box plot evidencing raw counts obtained from healthy donor EV samples using both triple-coated or CD61 beads. A statistically significant difference between groups could be appreciated (p-value = 0.00053). (B) Box plot evidencing raw counts obtained from healthy donor and patient EV samples using CD61 beads. No statistically significant difference could be found between groups (p-value = 0.43). [file 40364_2022_404_MOESM4_ESM.pdf]

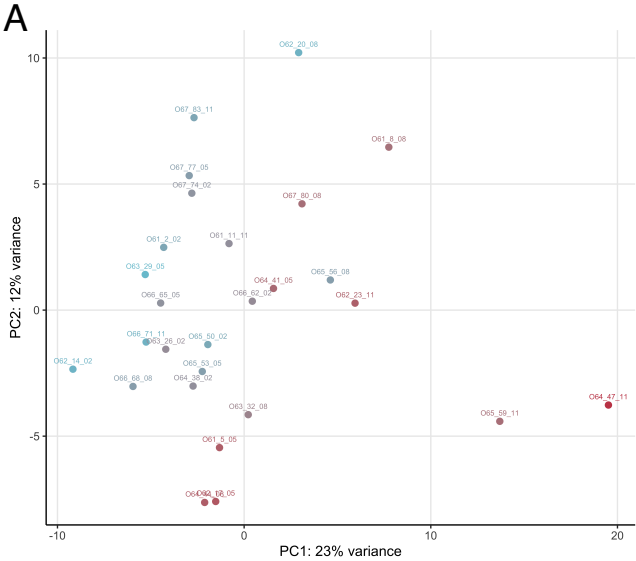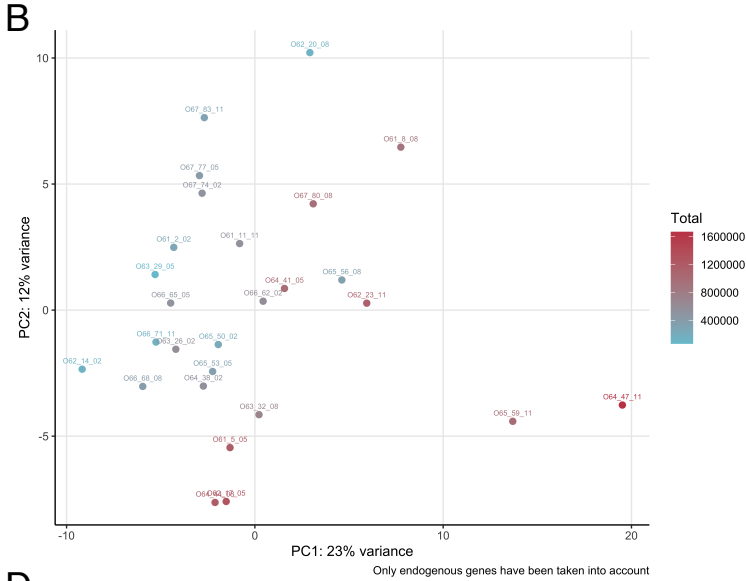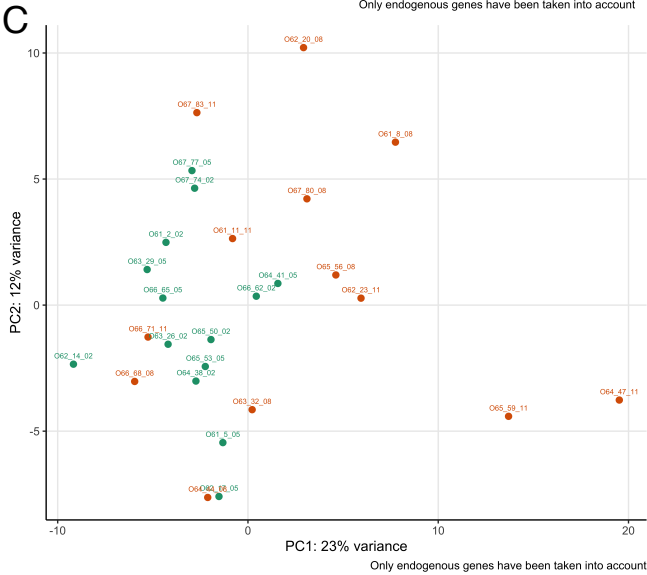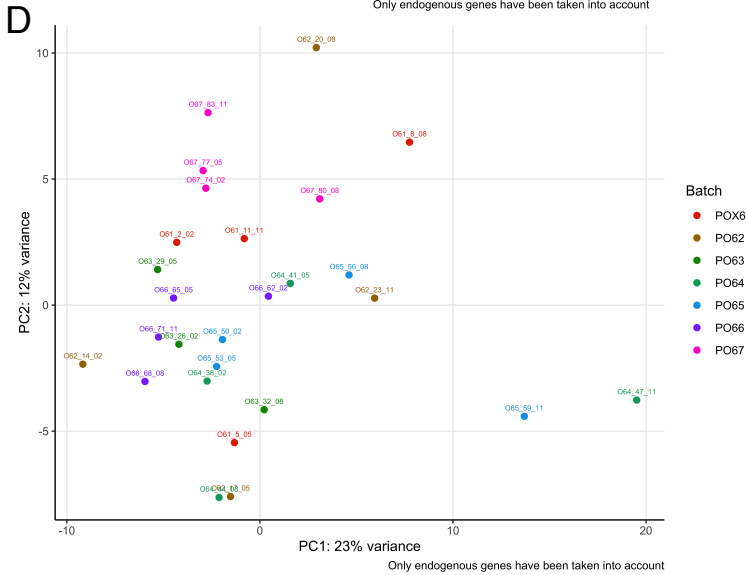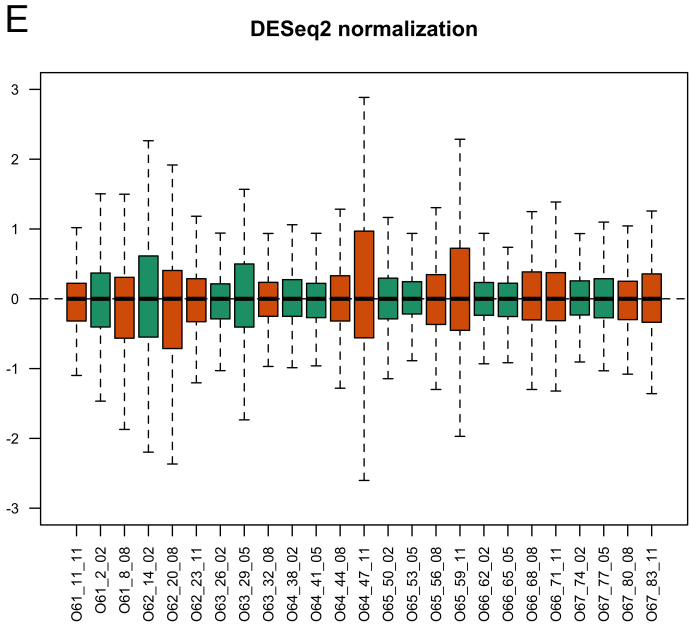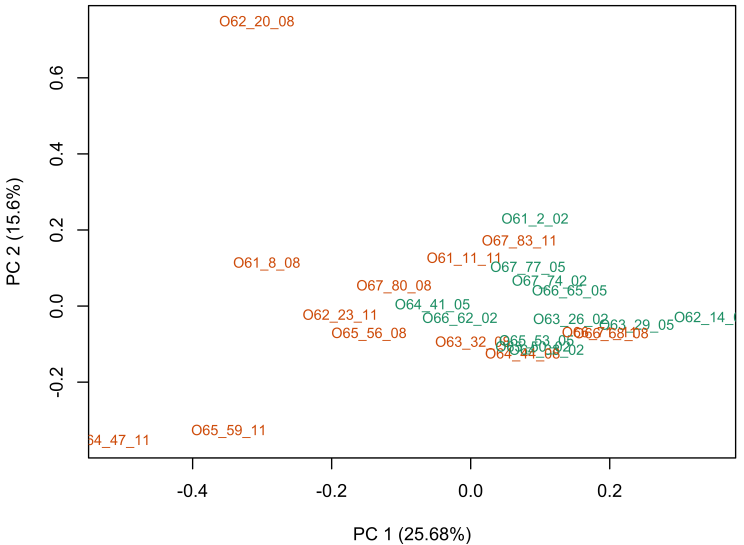

Supplement: Supplementary file 5 — Additional file 5: Supplementary Figure 5. EDA and normalization of gene expression data comparing two platelet-derived EV datasets obtained from healthy donors and early-stage NSCLC patients. (A to D) Principal component analysis (PCA) was performed on unnormalized gene counts for exploratory data analysis. Principal components PC1 and PC2 were plotted on the X and Y axis, respectively. Different variables are evidenced:(A) Gene counts excluding internally-defined nCounter control genes, (B) Total read count per sample, (C) Sample groups representing the two cohorts compared, healthy donors vs. early-stage NSCLC, (D) Due to a maximum number of 12 slots per nCounter experiment, samples were processed in different batches. Each batch represents one individual nCounter run. (E) Relative log expression (RLE) plots for visualization of the normalization performance with DESeq2 (left). Normalized count data was projected on PC1 and PC2 after PCA (right). Healthy donor and early-stage NSCLC patient samples were represented in green and orange, respectively. [file 40364_2022_404_MOESM5_ESM.pdf]
