# Supplementary material for: Selective isolation of extracellular vesicles from minimally processed human plasma as a translational strategy for liquid biopsies
Source: Biomark Res. 2022 Aug 7;10:57. doi: 10.1186/s40364-022-00404-1 (PMC9357340; doi:10.1186/s40364-022-00404-1)
Supplement: Supplementary file 6 — Additional file 6: Supplementary Table 1. Quantification of bead input and EV spikes used across IP experiments. We assured that beads always exceeded the number of EVs, but also avoided an overabundance of the former. [file 40364_2022_404_MOESM6_ESM.pdf]

Figure	Beads	Target	Input (particle number)
1A	MACS-STV	CD9	1,84E+09
		ISO	1,46E+09
	MACS	CD9	5,50E+09
		CD61	2,46E+09
	**Spike: HEK293-CFSE**		5,15E+08
1C	MACS-STV	CD9	1,61E+09
		ISO	1,18E+09
	MACS	CD9	5,50E+09
		CD61	2,46E+09
	**Spike: HEK293-CFSE**		3,76E+08
1E	MACS	CD9	6,05E+09
		PE	6,75E+09
	**Spike: HEK293-CFSE**		4,91E+08
2B	MACS	CD9	2,12E+10
		PE	3,27E+10
	**Spike: 22RV1-NIR**		2,82E+08
2C	MACS	CD9	2,12E+10
		PE	3,27E+10
	**Spike: 22RV1-NIR + CD9-PE**		7,86E+07
3A	MACS CD9 + CD63 + CD81		1,42E+10
	**Spike: 22RV1-NIR**		1,83E+08
	**Spike: HT29-CFSE**		5,01E+08
3B	MACS CD9 + CD63 + CD81		1,42E+10
	**Spike: HT29-CFSE**		2,00E+08
	**Spike: HEK293-CFSE**		2,00E+08
	**Spike: A549-CFSE**		2,00E+08
3C	MACS CD9 + CD63 + CD81		1,42E+10
	**Spike: HEK293-CFSE**		4,00E+08
	**Spike: HT29-CFSE**		4,00E+08
